# Modelling Hotspots for Invasive Alien Plants in India

**DOI:** 10.1371/journal.pone.0134665

**Published:** 2015-07-31

**Authors:** Dibyendu Adhikari, Raghuvar Tiwary, Saroj Kanta Barik

**Affiliations:** Department of Botany, North-Eastern Hill University, Shillong-793022, Meghalaya, India; University of New South Wales, AUSTRALIA

## Abstract

Identification of invasion hotspots that support multiple invasive alien species (IAS) is a pre-requisite for control and management of invasion. However, till recently it remained a methodological challenge to precisely determine such invasive hotspots. We identified the hotspots of alien species invasion in India through Ecological Niche Modelling (ENM) using species occurrence data from the Global Biodiversity Information Facility (GBIF). The predicted area of invasion for selected species were classified into 4 categories based on number of model agreements for a region i.e. high, medium, low and very low. About 49% of the total geographical area of India was predicted to be prone to invasion at moderate to high levels of climatic suitability. The intersection of anthropogenic biomes and ecoregions with the regions of 'high' climatic suitability was classified as hotspot of alien plant invasion. Nineteen of 47 ecoregions of India, harboured such hotspots. Most ecologically sensitive regions of India, including the 'biodiversity hotspots' and coastal regions coincide with invasion hotspots, indicating their vulnerability to alien plant invasion. Besides demonstrating the usefulness of ENM and open source data for IAS management, the present study provides a knowledge base for guiding the formulation of an effective policy and management strategy for controlling the invasive alien species.

## Introduction

Invasive alien species (IAS) pose serious environmental problems by disassembling natural communities, and adversely affecting ecosystem structure and function [1–4]. The negative impacts of IAS on native biodiversity, economy, and human health are well-established [5, 6]. Major drivers of global change such as climate warming, deforestation, habitat fragmentation, changes in land use and land cover, rapid economic development, and population explosion accelerate the invasion process [4]. Several different approaches have been taken to the study of alien species in India during the last two decades. These include floristic survey and documentation, ethnobotanical description, and experiments in the disciplines of ecology, ecophysiology and genetics [4, 7–18]. However, most of these studies were conducted at a local scale, and are species-specific, providing limited scope to construct a pan-Indian IAS scenario. In general, research efforts on alien species invasion in the Indian sub-continent are far behind in scope, intensity and scale compared to other regions of the world [12, 19]. Considering the high taxonomic diversity, rapid spread and large distribution area of IAS in India, alien species invasion has not attracted an appropriate level of attention of the scientific and policy making communities. In order to develop an effective strategy for control of IAS, construction of a macro-level comprehensive spatial picture involving maximum possible number of invasive plants in the region is a pre-requisite [20, 21]. Delineating the areas climatically suitable for invasion by diverse species would facilitate formulation of appropriate policy for their control and management [22].

In this paper, we propose to delineate areas suitable for multiple IAS in India and define 'invasion hotspots' for prioritization and management of IAS. We consider ‘invasion hotspots’ as, ecoregions with more than 50 percent of their area climatically suitable for introduction, establishment, and persistence of multiple invasive alien species, and have significant human influences. The phrase ‘multiple invasive alien species’ is used in this paper to indicate the 'presence' and/or 'potential of occurrence' of more than one species in a given region. The concept of 'invasion hotspot' in this paper is primarily inspired from Myers' concept of ‘biodiversity hotspot’ [23]. The term 'invasion hotspot' was earlier used by O'Donnell et al. [24] for regions having high potential for invasion by diverse invasive alien species.

We identified the hotspots of alien plant invasion by intersecting the areas climatically suitable for multiple IAS with the ecoregions and anthropogenic biomes. To identify climatically suitable areas for IAS, Ecological Niche Modelling (ENM) based on the realized niche concept of the species was used [24], where species distribution is interpolated and extrapolated in a geographic space [25, 26]. ENM delineated the bioclimatic envelope of each species which constitutes the climatic component of the fundamental ecological niche, or ‘the climatic niche’ [27, 28]. The climatic niches of the multiple IAS were used to delineate the hotspots because of the following reasons: (i) climate exerts dominant control over the natural distribution of species, which is the central premise of biogeography [22, 29, 30], (ii) the climatic niche has been proved to be important in predicting species distribution at large spatial scales i.e. regional, continental and global [30], (iii) climatic niches of invasive species are conserved i.e. do not differ between the native and invasive range populations [31], and (iv) high predictive performance of climatic niche models on species invasion at coarse spatial resolution [22, 24] demonstrates primacy of climate over bionomic variables i.e. dynamically consumed variables for which competition occurs [32].

Our approach is based on the recognized fact that the basic requirement for successful invasion is the climatic similarity between the native and projected regions [33, 34]. This has further been confirmed with evidence that establishment and spread of alien plants are limited by an imperfect climate match [35]. Therefore, climate matching approach has been used by several workers for predicting species distribution [22, 24, 27] and invasion risk of IAS [20, 36]. However, the role of several other non-climatic factors such as geology, soil, disturbance regime, land use changes, competition, proximity to sites of introduction, and other biotic interactions determining the presence or absence of a species in a particular area cannot be denied. The patterns of alien plant invasion in fact are a function of scale, community–environment relationships, spatial autocorrelation effects, and species-specific responses to environmental perturbations including barriers to dispersal. However, several workers have prioritized climate matching over other factors in the context of plant invasion [37].

## Materials and Methods

### Selection of IAS, ecoregion and anthropogenic biome

Initially, 295 invasive plant species of alien origin occurring in India were selected based on their wide ecological amplitude and the severity of impacts on the ecosystems in which they occur. These 295 species were checked for availability of georeferenced global occurrence records for model training, and accordingly 155 species were selected. Species occurrence data from Global Biodiversity Information Facility (GBIF) were used for model building. Of the total 825 global terrestrial ecoregions [38], 47 ecoregions located within the political boundary of India were selected. Similarly, of the 35 global biodiversity hotspots [39], 4 hotspots in India were selected viz., Himalayas, Western Ghats, and parts of Indo-Burma and Sundaland. All the 21 anthropogenic biomes under six groups [40] were included in the analysis for delineation of invasion hotspots.

### Geographical extent of analysis

**Fig 1:**
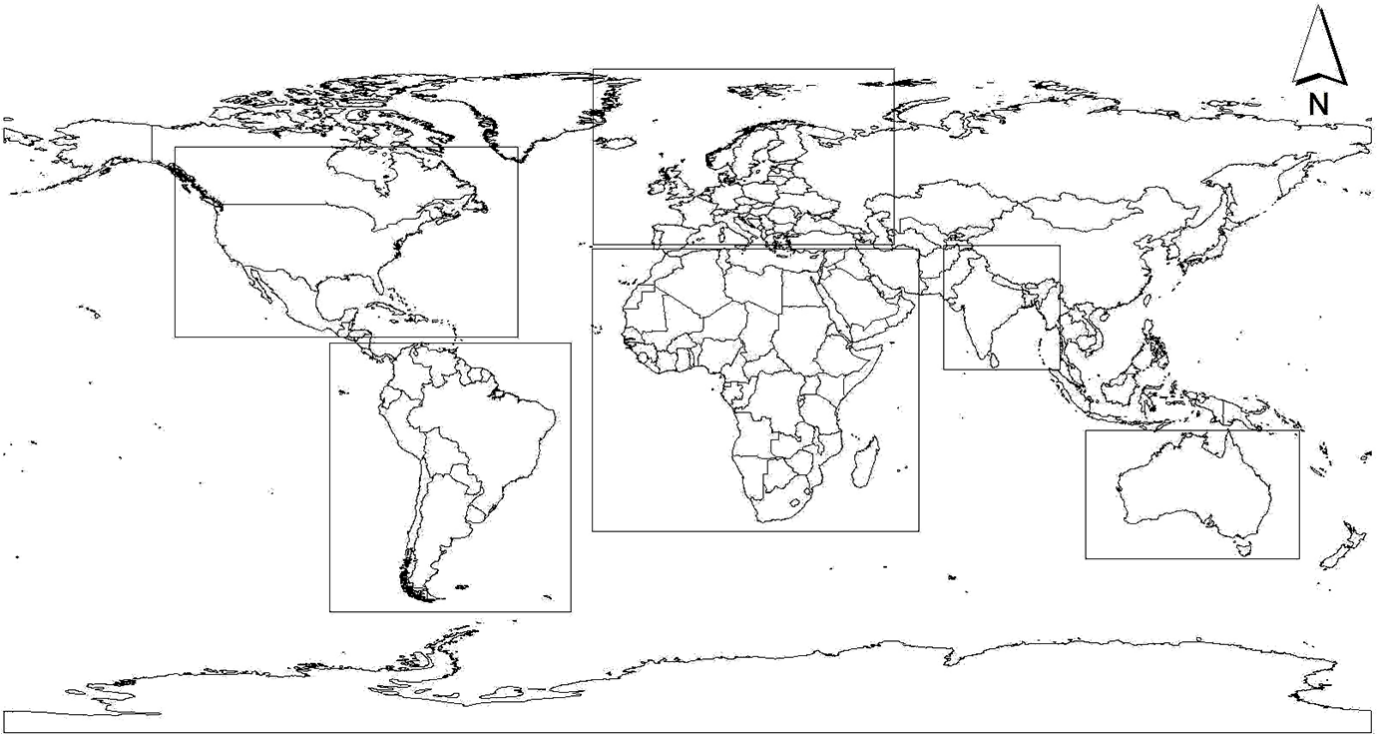
Map showing the bounding boxes used to delimit the geographical extent of species occurrence data and bioclimatic data used for model calibration in the training ranges of North and South America, Europe, Africa and Australia; and the bioclimatic data for the projected range in the Indian region.

We selected Africa, Australia, Europe, North America and South America as training regions for the niche models (Fig 1). Data from species’ entire distribution ranges provide a closer approximation of the fundamental niche [41]. Therefore, occurrence data points spread over a large geographic region e.g., at continental level, represent adequate sampling extent and encompass broad range of environmental conditions in which the species occurs. These two conditions are essential for robust distribution modeling of the IAS [42]. The modelled bioclimatic niches were then projected to India with a total geographical area of 3,166,414 km^2^.

### Delineating invasion hotspots

We delineated the hotspots of invasion in the framework of modelled climatic suitability, ecoregional setting, and anthropogenic influences. Identification of climatically suitable areas for each IAS is fundamental to hotspot delineation [22]. An ecoregion comprises a geographically distinct set of natural communities, sharing a majority of the species, ecological dynamics, environmental conditions, and critical ecological interactions important for their long-term persistence [43]. Since these communities function together as a single conservation unit at regional scale and the impact of IAS invasion on them would be very similar, inclusion of ecoregion as a component for hotspot delineation would facilitate conservation planning. Anthropogenic biomes were considered as the third criterion for hotspot delineation because human influences facilitate the invasion process [2]. Anthropogenic biomes are the result of sustained and direct human interaction with ecosystems, and were identified through empirical analysis of global population, land use, and land cover [40]. Thus, the consensus zone of the ecoregions, anthropogenic biomes, and areas with high climatic suitability for multiple alien invasive plant species represented a hotspot.

### Bioclimatic data and grain size

Climate plays a major role in determining the distribution of plants [44–46]. We used 19 bioclimatic variables relevant to persistence, phenology and physiology of the selected species to model the climatic niche (Table 1). The bioclimatic variables are derivatives of monthly temperature and precipitation values. These variables with a spatial resolution of 2.5 arc minutes were downloaded from the worldclim website (www.worldclim.org).

**Table 1:** Environmental variables used for modelling and delineating invasion hotspots.

Environmental data	Source
**Bioclimatic data**	www.worldclim.org
Bio1—Annual Mean Temperature	
Bio2—Mean Diurnal Range (Mean of monthly (max temp—min temp))	
Bio3—Isothermality (Bio 2/Bio 7) (* 100)	
Bio4—Temperature Seasonality (standard deviation *100)	
Bio5—Max Temperature of Warmest Month	
Bio6—Min Temperature of Coldest Month	
Bio7—Temperature Annual Range (Bio 5—Bio 6)	
Bio8—Mean Temperature of Wettest Quarter	
Bio9—Mean Temperature of Driest Quarter	
Bio10—Mean Temperature of Warmest Quarter	
Bio11—Mean Temperature of Coldest Quarter	
Bio12—Annual Precipitation	
Bio13—Precipitation of Wettest Month	
Bio14—Precipitation of Driest Month	
Bio15—Precipitation Seasonality (Coefficient of Variation)	
Bio16—Precipitation of Wettest Quarter	
Bio17—Precipitation of Driest Quarter	
Bio18—Precipitation of Warmest Quarter	
**Ecoregion**	www.maps.tnc.org/
**Anthropogenic biome**	www.sedac.ciesin.columbia.edu/

Selection of an appropriate grain size for environmental variables and species occurrence data is important for enhancing the prediction accuracy in ENM [47–50]. We resampled the predictor layers to a spatial resolution of 0.04° (≈ 4km), and ran the models at a relatively coarser scale because: (i) the geographic scale of species distribution was quite large, (ii) occurrence data for a large number of species were used, (iii) number of occurrence data points varied widely among the species, (iv) interpolated climatic variables were used as predictors, (v) there was a possibility of mismatch between species point data and the corresponding environmental raster data, (vi) possibility of spatial autocorrelation in the environmental data, and (viii) constraints of computation time and resources.

### Species occurrence data

About two million global distributional records for the selected 155 species were downloaded from the web resource of the Global Biodiversity Information Facility (www.gbif.org) based on: (i) invasiveness in the subcontinent, and (ii) availability of adequate and well-spread geographical coordinates. These records were checked for spatial errors using Diva-GIS [51]. For each species, only one occurrence per 0.04° grid cell was retained and the multiple occurrences per cell were removed using ENM Tools 1.3 [52]. Finally, 239,236 occurrence records were used in model calibration, evaluation and projection (S1 Table **)**. Presence of these species in India was confirmed through sample field surveys, available literature [13, 16, 17], and from online resources viz., Global Invasive Species Database (www.issg.org) and Global Species website (www.globalspecies.org).

### Model calibration and thresholding

The climatic niche of each selected species was modelled using maximum entropy ecological niche modelling software MaxEnt 3.3.3k [42]. MaxEnt estimates the maximum entropy probability distribution function based on the input environmental variables in order to predict the area of potential distribution of a species. It defines the ecological niche boundary of the species by constraining the probability distribution based on the environmental parameters of the grid-cell presence record [42]. The attributes of MaxEnt making it the most widely used software for ecological niche modelling are: a strict mathematical definition, continuous probabilistic output, simultaneous handling of continuous and categorical environmental data, functionality to investigate variable importance through jackknife procedure, capacity to handle low sample sizes, facility to allow cross-validation, bootstrapping and repeated subsampling in order to test model robustness, multivariate environmental suitability surfaces tool to identify novel climate conditions, and simplicity of model interpretation [42, 53–55].

The Maxent model was run with default settings for the following parameters: number of background points used (10,000), number of iterations (500), convergence threshold (0.00001), and regularization multiplier (1). We selected the 'auto features' option in Maxent software as it helps in selecting the appropriate model fitting functions in respect of each of the species. To derive the optimized model, we executed 10 replicated runs for each species by employing a cross validation technique. In cross validation, the occurrence data is randomly split into a number of folds. Models are generated leaving out each fold in turn, and the left out folds are used for intrinsic evaluation [56]. Cross validation technique was followed to optimize the model by executing 10 replicated runs that generated average, maximum, minimum, median and standard deviation. Model quality was evaluated based on AUC value, and the models were graded as poor (AUC<0.8), fair (0.8<AUC<0.9), good (0.9<AUC<0.95), and very good (0.95<AUC<1.0) [57]. The probabilistic models were truncated by applying the 10 percentile training presence logistic threshold rule in MaxEnt, which generated species-specific binary maps of climate suitability and unsuitability [58]. Novel climatic conditions in India compared to the model training continents were identified and analysed using ExDet Tool Ver. 1.0 [54]. ExDet (Extrapolation Detection) is a robust tool which helps in detecting and quantifying the environmental novelties i.e. novel univariate range (Type 1 novelty) and novel combinations of covariates (Type 2 novelty), in the projected landscapes.

### Multispecies climatic suitability maps and identification of invasion hotspots

Multispecies climatic suitability maps were generated by summing up fair, good, and very good quality thresholded models in ArcGIS platform. These climate suitability maps comprised six different sets of consensus models characterized by different degrees of model agreement. In a given consensus model, areas having maximum model agreements were considered suitable for occupancy by maximum number of IAS. In total six consensus maps were generated, of which five maps depicted the projections for Africa, Australia, Europe, North America and South America, and the sixth map depicted a generalized picture of climate suitability for IAS. The sixth map was generated by summing up the multispecies projections of all five model training continents. The species-specific models for each of the 5 continents (as per availability), that represented a 'climate information bit' were combined to obtain the climatic niche of all 155 IAS. Summing-up of these information bits in the projected landscape yielded a consensus map of 525 models with a range of model agreements (15–284 model combinations) which were subsequently categorized as high, medium, low and very low through quantile classification in ArcGIS. The region with the maximum agreements was designated as 'high' category, and was deemed to be suitable for a large number of IAS. The alternate technique for the above classification could have been to use global occurrence data of each species for species-wise projection models and subsequent generation of a consensus map. However, given the fact that most of the species selected were not uniformly distributed throughout the world, such an approach would have resulted in environmental bias. This bias arises out of the spatial bias caused due to collection of non-random occurrence data and background sampling [59].

The classified climatic suitability maps were then overlaid on the terrestrial ecoregions [43], (The Nature Conservancy, http://maps.tnc.org/gis data.html), and the anthropogenic biomes [40], (CIESIN: http://sedac.ciesin.columbia.edu). The ecoregions where >50 percent of the area is climatically suitable with a 'high' consensus level, and contain at least three anthropogenic biome types, were considered as 'invasion hotspots'. The identified invasion hotspots were overlaid on biodiversity hotspot shape files (available at www.conservation.org) using ArcGIS software, to identify the areas vulnerable to alien species invasion. A KML version of the invasion hotspots was created using DIVA GIS 7.5 [51], which was then superimposed on Google Earth 7.1.2 to identify vulnerable forest areas.

## Results

### Species selection

The selected 155 IAS belonged to 46 families. Asteraceae (23) had the highest number of species followed by Fabaceae (15), Euphorbiaceae in the conventional sense (11), Amaranthaceae (10) and Convolvulaceae (10). Of these, 79 species were annuals, 72 perennials, and 4 were biennials. These species represented various stages of invasion viz., colonization, establishment and spreading in India, and belonged to diverse life-forms such as trees, shrubs, herbs, climbers, grasses, sedges and succulents (S1 Table **)**. Among all the life-forms, herbs had maximum species (102 species—61 annuals, 4 biennials, and 37 perennials) followed by shrubs (25), climbers (13), grasses (7), sedges (3), trees (3) and succulents (2).

### Model performance

**Fig 2:**
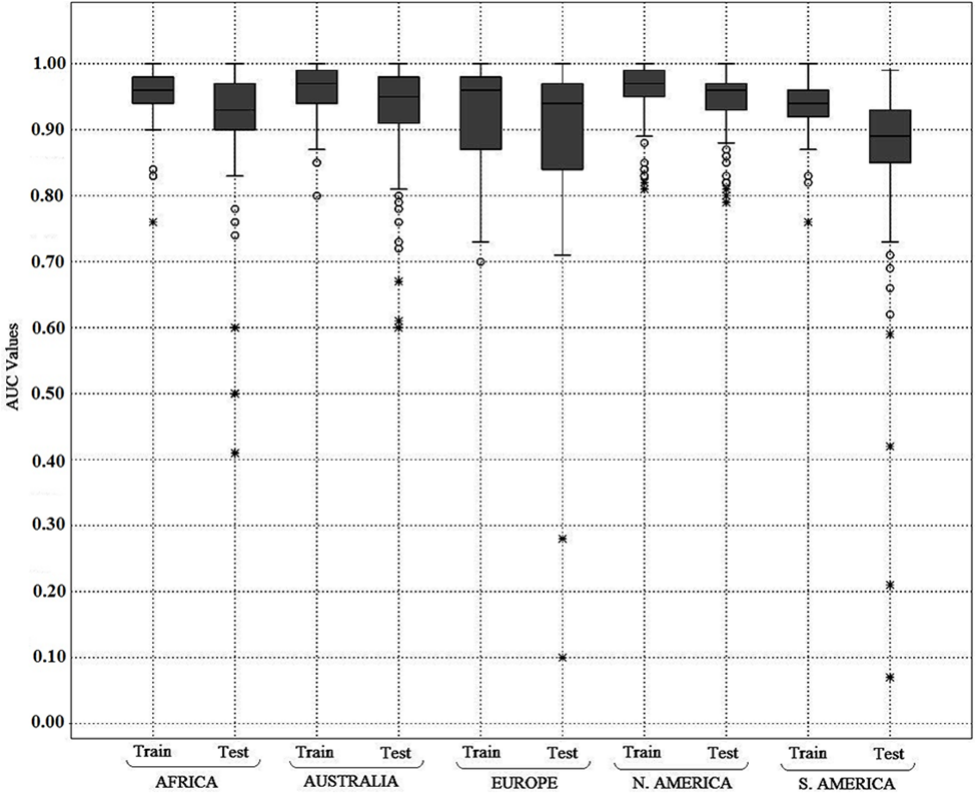
Box plot showing distribution of mean AUC_train_ and AUC_test_ values for the selected alien invasive plants in each of the model training continents. In the figure, the boxes represent 25–75 percent quartiles of the AUC values, and the horizontal line inside the boxes represents the median. The minimal and maximal values are shown with whiskers drawn from the top of the box up to the largest data point less than 1.5 times the box height from the box, and similarly below the box. The circles represent the outlier values, and the stars represent the extreme values.

Based on the AUC values, species-specific models from each of the continents were graded as very good, good, fair, and poor. More than 85 percent of the AUC_train_ and AUC_test_ values for all the continents occurred in the AUC range of 0.8 to 1.0 that discriminated the suitable and unsuitable climates at fair, good and very good levels (Fig 2, Table 2 **)**.

**Table 2:** Consolidated result showing the number of species categorized under various model grades on the basis of species-specific mean AUC_train_ and AUC_test_ values in the respective training ranges. Grading of model quality was done based on AUC value as: Poor (<0.8), Fair (0.8–0.9), Good (0.9–0.95) and Very good (0.95–1.0).

Continent	Number of species under various model grades based on mean AUC_train_				Number of species under various model grades based on mean AUC_test_			
	Very good	Good	Fair	Poor	Very good	Good	Fair	Poor
Africa	87	41	3	1	56	45	23	8
Australia	91	17	13	0	64	32	17	8
Europe	36	8	8	8	30	6	14	10
North America	102	15	12	0	86	28	14	1
South America	61	56	8	1	22	31	57	16

### Climate novelty

The ExDet tool detected maximum climatic novelty (Type 1) in India for bioclimatic background projections of Europe and South America, while relatively less novelty was detected in the projections from Africa, Australia and North America (S1 Fig). Though most regions in India have 'Type 1' climatic novelty, novel combination of bioclimatic variables i.e. Type 2 novelty, was detected in some areas of Western Ghats, northeastern India and Western Himalaya (S1 Fig). The climatic novelty in these area was due to temperature and precipitation seasonality (S2 Fig).

### Climate suitability and invasion hotspots

**Fig 3:**
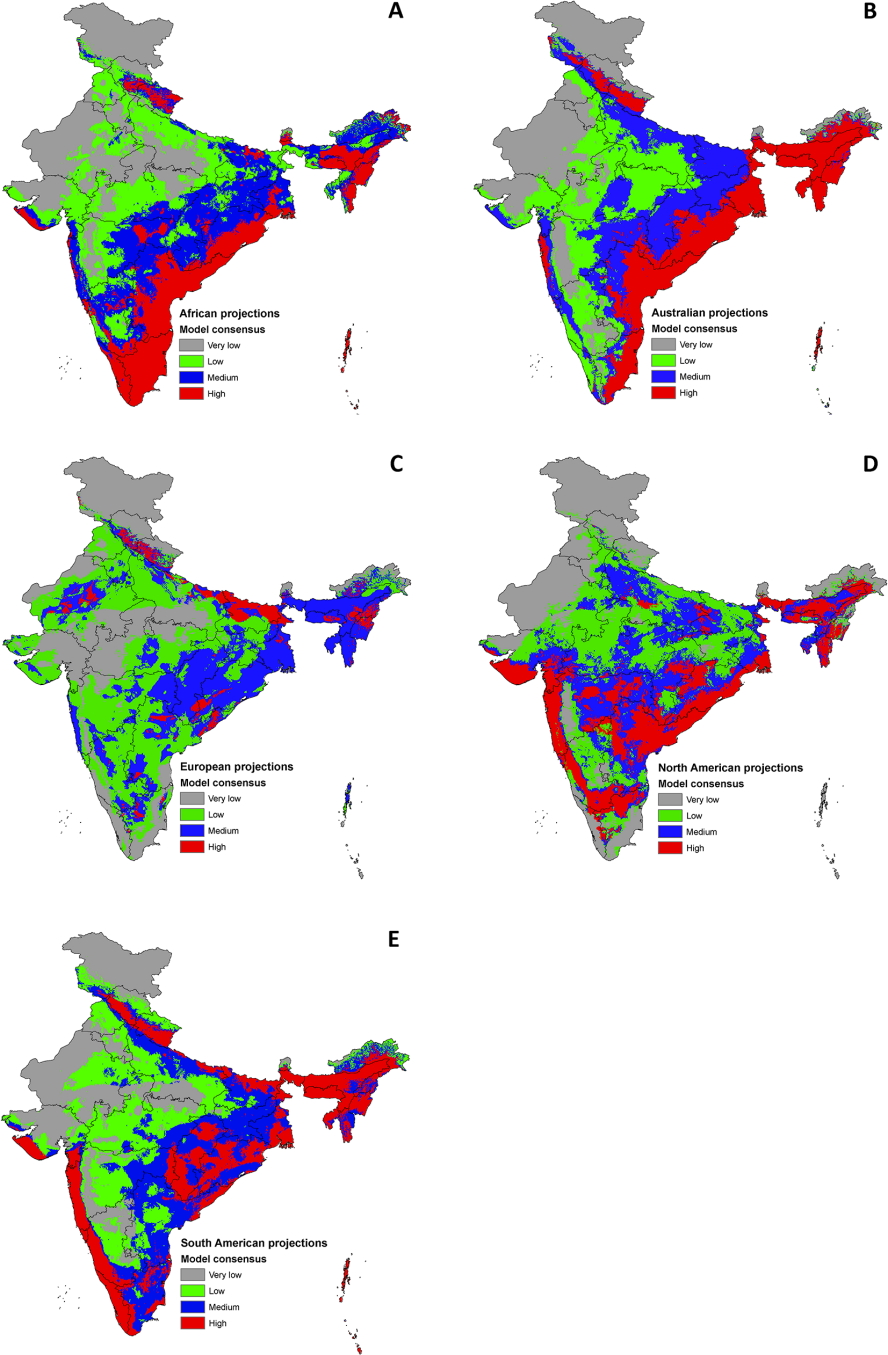
Climatic suitability map for diverse alien plant species in India based on continent of origin. A-E show the model consensus identified by summing up the thresholded model projections for multiple species from—(A) Africa, (B) Australia, (C) Europe, (D) North America, and (E) South America. The figure legend show the model agreements, wherein ‘high’ represents the areas with highest model agreement, and ‘very low’ having the lowest agreements. Higher model agreements represent larger number of alien invasive species while the lower model agreements represent lower number of alien invasives.

Continent-specific niche model projections depicted varied pictures of climatic suitability in India. Projections from Africa and Australia mostly predicted the northeastern region and eastern coasts of Peninsular India to have 'high' climate suitability. The North and South American projections matched with the peninsular and northeastern region of India. Western Himalaya was predicted to have 'high' climate suitability with the projections from South America. However, projections from Europe presented a slightly discordant picture with areas of 'high' climatic suitability in the Western Himalaya, Terai areas bordering India and Nepal, Aravalli range in Rajasthan, Hills in the Eastern Ghats bordering Andhra Pradesh and Odisha, and the Naga and Khasi Hills in northeastern India (Fig 3).

**Fig 4:**
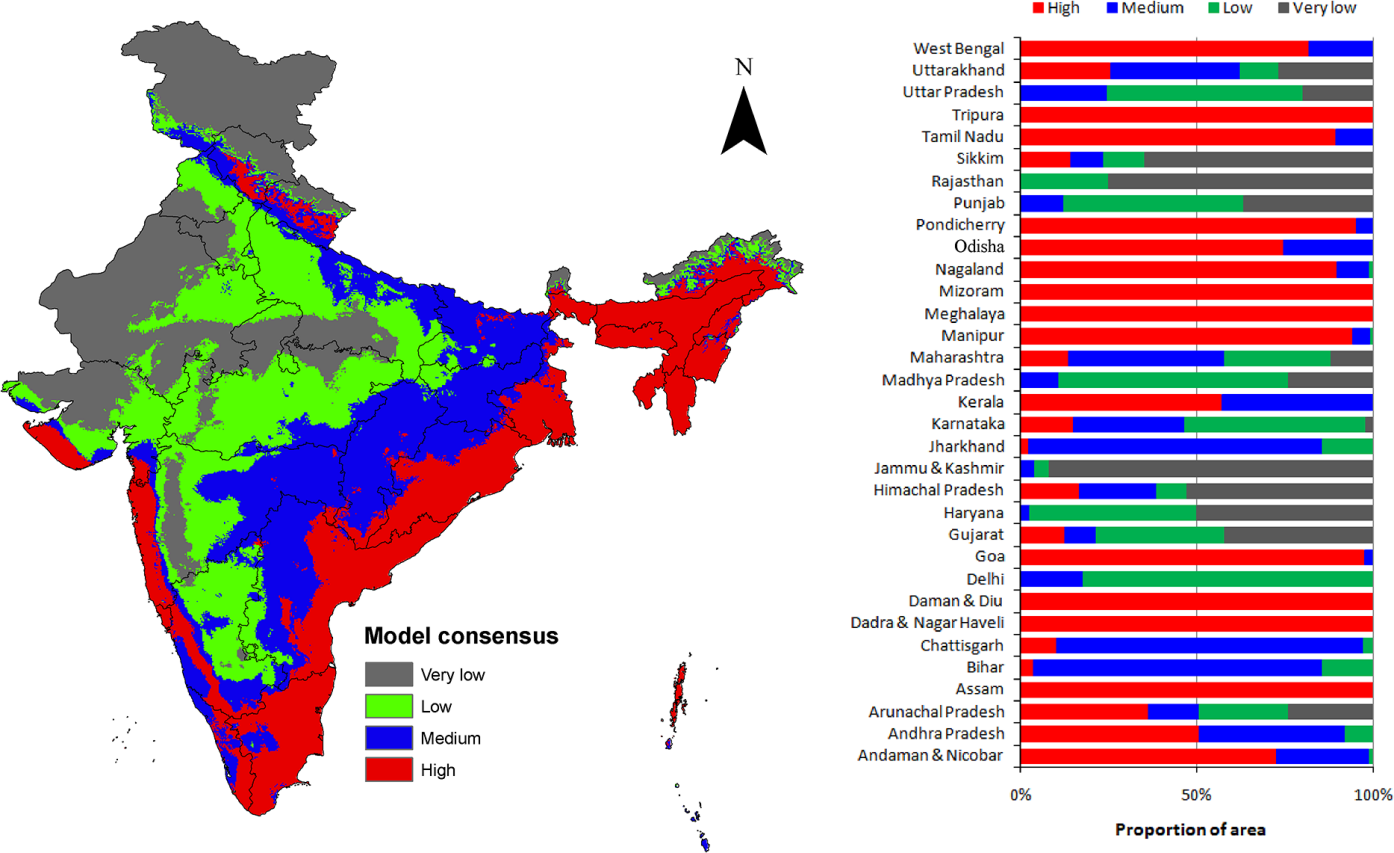
Model consensus classes depicting climatic suitability for diverse IAS in India. The bar diagram depicts the percentage of total geographical area under various consensus class in different states of India. The climate suitability map was generated by combining 525 thresholded model projections from Africa, Australia, Europe, North America and South America. The legend show the model agreements, wherein 'high' represents the areas with highest range of model agreements, and 'very low' having the lowest range of model agreements. 'High' agreement represent high species richness, while 'low' agreement represent low species richness.

The combined projection from all the 5 continents revealed that the coastal areas, the northeastern region, and the Western Himalaya have 'high' climatic suitability for diverse IAS. The projections reveal that more than half of Andaman and Nicobar Islands, Andhra Pradesh, Assam, Dadra and Nagar Haveli, Daman and Diu, Goa, Kerala, Manipur, Meghalaya Mizoram, Nagaland, Odisha, Pondicherry, Tamil Nadu, Tripura and West Bengal, have 'high' climatic suitability for IAS (Fig 4).

Overlaying the combined projection on the ecoregions and major biome types in India revealed their potential to harbor diverse IAS (S2 Table, S3 and S4 Figs). Out of the 47 ecoregions in India, 29 have at least 10% of their total area under 'high' model consensus (S4 Fig). The ecoregions that have proportionately large areas i.e. >90% under the 'high' consensus level are: (1) Andaman islands rain forest, (2) Brahmaputra valley semievergreen forests, (3) Chin-Hills-Arakan Yoma montane forests, (4) East Deccan dry-evergreen forests, (5) Godavari-Krishna mangroves, (6) Meghalaya subtropical forests, (7) Mizoram-Manipur-Kachin rainforests, (8) Orissa semievergreen forests, (9) Sunderbans mangroves, and (10) Sunderbans freshwater swamp forests. Of the 11 major biome types, the most vulnerable to species invasion are: (1) tropical and subtropical moist broadleaf forests, (2) tropical and subtropical dry broadleaf forests, (3) tropical and subtropical grasslands, savannas and shrublands, (4) tropical and subtropical coniferous forests, (5) temperate broadleaf and mixed forests, and (6) mangroves. These biomes may support 'moderate' to 'high' richness of IAS. However, biome types such as Flooded grasslands and savannas, Montane grasslands and shrublands, Rock and ice, and Temperate conifer forests, have more areas under 'very low' consensus class, and hence have less chances of supporting diverse IAS. Nineteen out of 47 ecoregions showed 'very low' climatic suitability for persistence of diverse IAS (S4 Fig).

**Fig 5:**
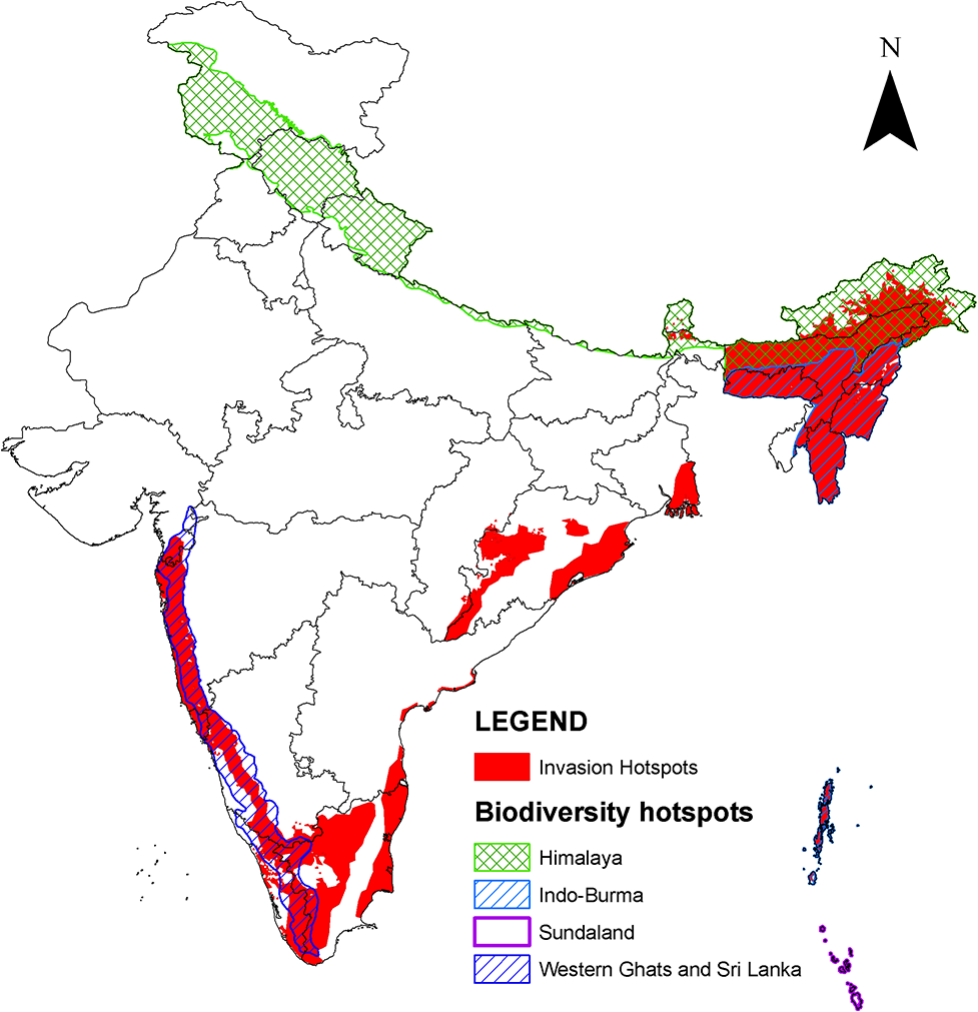
Invasion hotspots in India delineated through intersection of ecoregions, multi-species ecological niche model consensus, and anthropogenic biomes. The delineated areas cover >50 percent area of various ecoregions, are climatically suitable for a large number of invasive alien species, and have signature of diverse anthropogenic activities. The boundary files on biodiversity hotspots were overlaid on the modelled invasion hotspots matches to portray the level of threat to these areas from invasive alien species.

**Fig 6:**
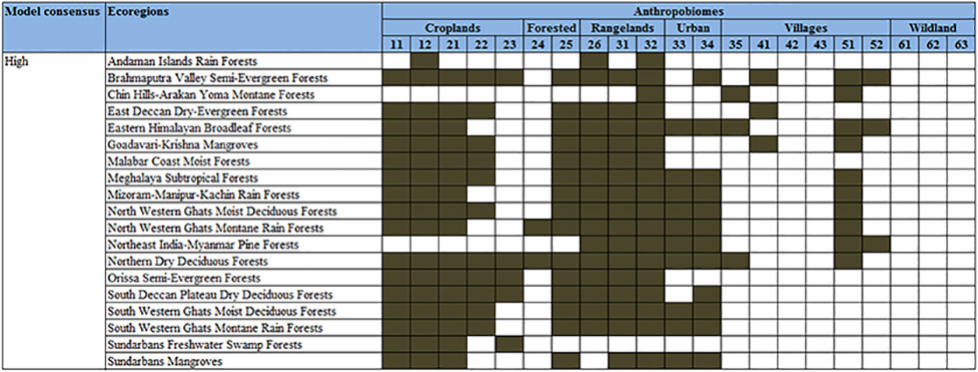
Matrix depicting the 3 components viz. climatic suitability, ecoregions and anthropogenic biomes, used for delineation of invasion hotspots in India. The identified ecoregions have > 50 percent of its area under the high climatic suitability. The numbers on top of the matrix indicate the major categories of anthropogenic biomes: Croplands: (11) Residential irrigated cropland, (12) Residential rainfed mosaic, (21) Populated irrigated cropland, (22) Populated rainfed cropland, (23) Remote croplands; Forested: (24) Populated forests, (25) Remote forests; Rangelands: (26) Residential rangelands, (31) Populated rangelands, (32) Remote rangelands; Urban: (33) Urban, (34) Dense settlements; Villages: (35) Rice villages, (41) Irrigated villages, (42) Cropped & pastoral villages, (43) Pastoral villages, (51) Rainfed villages, (52) Rainfed mosaic villages; Wild Land: (61) Wild forests, (62) Sparse trees, (63) Barren. Description of the anthropogenic biomes is given in Ellis and Ramankutty (2008), and details on ecoregion-wise information can be viewed at http://www.globalspecies.org/ecoregions. The filled boxes signify representation of the particular anthropobiome in each ecoregion.

The identified invasion hotspots coincide the major biodiversity hotspots of India i.e. Western Ghats, Indo-Burma, and Eastern Himalaya (Fig 5). They contain diverse signature of human activities, and are characterized by 5 major anthropogenic biome types and 18 sub-types (Fig 6). Apart from the biodiversity hotspots, the invasion hotspots also coincide forest reserves, islands, coastal forests and mangrove ecosystems of India.

## Discussion

By using a climate-based approach, recent research has identified species having invasion potential in climatically similar regions [20, 22, 24, 31]. A synthesis of data on the characteristics of invasive species and identification of correlates of invasion within and across different biological groups also revealed that climate/habitat match is the only characteristic that is significantly and consistently associated with invasive behaviour across biological groups including plants [37]. While the utility of climatic niche at large spatial scales such as regional, continental and global is well-established, the role of other non-climatic factors in determining the presence or absence of a species at finer i.e. regional or local scales is also supported by many studies [33, 34]. Climate principally affects the distribution of invasive species as they can only succeed in regions where they are not limited by climatic constraints. Moreover, the conservatism of climatic niche makes the climate-based model predictions widely applicable [53]. Considering the above facts and in view of the major advantage of ENM in assessing the invasion potential of large numbers of species, in the present study, climatic niches of the multiple IAS were modelled [60].

Comprehensive distribution maps depicting climatic suitability and invasion hotspots in India were generated through niche models of a broad spectrum of species covering different taxa, life-forms, and plant functional types. The continent-specific multispecies ENM projections show some discordance in the scenarios of climatic suitability for IAS in India (Fig 3). Such discordance may principally be attributed to variation in the ecological niche models resulting from the inbuilt correlation structure of the bioclimatic datasets of each continent [61]. Acknowledging the different sources of origin [13, 15, 16], building such scenarios would help us in having a better understanding of alien species invasion. Earlier workers reported that 35 percent of the alien plants in India are from South America, and the rest are from Asia (21%), Africa (20%), Europe (11%), Australia (8%) and North America (4%) [17]. Nevertheless, the continental projections showed some consensus areas that helped us in identifying the invasion hotspots in India. The model consensus clearly outlines the following:

### Invasion hotspots coincide with biodiversity hotspots

The concurrence of invasion hotspots and biodiversity hotspots may be attributed to similar environmental requirements of the alien species to that of their native counterparts [62]. Nevertheless, presence of an environmental gradient providing variability in resource availability may promote species invasion in the biodiversity hotspots [63–65].

The biodiversity hotspot regions in India have the highest concentration of the country's forest cover and include most of the protected areas [66]. In the last three decades, these biodiversity hotspots have experienced sizeable forest cover loss [67] because of biotic pressure, shifting cultivation, agricultural expansion, and urbanization [68]. Such disturbances create a state of disequilibrium in the environmental conditions, which make these areas vulnerable to invasion [69–73]. Such vulnerability of ecosystems to invasion may be explained based on the concept of availability of 'vacant niches' i.e. unfilled opportunities for additional species in ecosystems or by creating new 'ecological opportunities' for occupancy by the IAS [74, 75]. 'Ecological opportunities' denotes a pulse in the availability of readily assimilable nutrients in the novel habitats, essential for colonization, growth and development of IAS [76]. In fact, concurrence of invasion hotspots and the biodiversity hotspots is an indication of impending threat posed by the IAS to the global biodiversity hotspots. Human activities have been argued to facilitate species invasion [77, 78]. The identified invasion hotspots in this study also coincide areas with intense anthropogenic activities, which is evident from the dominance of anthropogenic biomes such as croplands, rangelands, urban areas, and rainfed villages (Fig 6). Thus, anthropogenic activities seem to be a strong correlate of invasion hotspots.

### Invasion hotspots coincide with islands, coastal forests, freshwater swamp forests, mangroves, and forest reserves

The invasion hotspots coincide with the Andaman Islands, the coastal forests, swamp forests, the mangrove ecosystems, and numerous forest reserves in India. The explanation for concurrence of these ecosystems with the invasion hotspots may be the same as mentioned above [62]. However, this concurrence should be viewed as an imminent threat to the native biodiversity of the islands, coastal forests, swamp forests, mangrove ecosystems, and forest reserves. The vulnerability of island ecosystems to alien species invasion has been attributed to the following reasons: (i) Islands have high net resource availability, and the native species have a poor ability to preempt those resources [76]. Consequently, the competitively superior invaders get an opportunity to occupy the vacant niches. (ii) Islands are exposed to high propagule pressure and the native species face a strong inter-specific competition from the alien invasive species [79], which make them susceptible to invasion. By and large, the above ecosystems are under constant threat of alien plant invasion due to landuse changes and biotic factors [80–82]. For example, although mangrove ecosystems in general are not susceptible to invasion, the mangrove forest areas adjoining terrestrial ecosystems, at the heads of estuaries, and areas having open canopy cover due to anthropogenic disturbances are susceptible to invasion. Some of the major port towns such as Mumbai, Ratnagiri, Panaji, Nagapattnam, Chennai, Kakinada, Paradip, and Haldia, fall within the identified invasion hotspots. Such areas may provide suitable habitats for colonization of IAS after being introduced through shipping routes [83]. Besides, the invasion hotspots also coincide with many hill and forest ranges, reserve forest areas, and protected areas in the Eastern Ghats, which have high floristic diversity. Some of the important forest areas and protected areas include: (1) Kanger valley forest area, (2) Ambapani sanctuary, (3) Karlapat wildlife sanctuary, (4) Kalahandi range, (5) Phulbani forest, (6) Gandhamardana hill reserve, (7) Malayagiri mountain range, (8) Harichandanpur-Telkoi reserve forest, (9) Khallikote reserve forest, (10) Chandaka elephant reserve, (11) Kapilash forest range, (12) Bhitarkanika national park, (13) Sunderbans national park, (14) Kamakhya forest range, (15) Redhakhol-Charmal range, (16) Malkangiri forest range, (17) Kalrayan hill forests, (18) Alangayam reserve forest, and (19) Jawadhu Polur reserve forest.

### Policy implications

Our work has illustrated the usefulness of open source data and software in generating a knowledge base having practical implications of IAS management by understanding and analyzing the alien species invasion problem which operates at a broad geographical scale. Some of the inferences that have strong policy implications are:

1. Invasion hotspots coincide with hotspots of biodiversity: This can be viewed from a threat perspective as it would endanger the persistence of many resident and endemic species. Enumeration of IAS should be undertaken in the biodiversity rich biomes and their impact on other native species at ecosystem level needs to be analysed.
2. Invasion hotspots coincide with islands, coastal areas and protected forest areas: Surveillance and quarantine measures should be stepped up in such areas as they may act as important sites of introduction, colonization and establishment of IAS.
3. Invasion hotspots coincide with croplands, rangelands and village biomes: Efforts for eradication and control of the alien invasives in these areas should be given top priority as it is relatively easier to control them at the beginning of their spread.
4. The remote forest biomes are unlikely to be invasion hotspots: The remote forest biomes did not coincide with any invasion hotspot. These biomes in India include areas with high tree cover and low human population. Therefore, it is important that such biomes are maintained in its present condition with minimum anthropogenic interferences.

### Caveats of the study

While recommending the above, we do recognize the following limitations of our predictions on hotspot of alien species: (i) although a biome may not support multiple IAS, yet a particular invasive species can pose serious threat to the biome, and (ii) the predictions are scale-dependent i.e. applicable to regional and global scales.

Extrapolating invasive species distribution models to a different geographical region relies on two fundamental assumptions of ENM: (i) species are at equilibrium with their surrounding environment, and (ii) they tend to retain their native range niche preferences in the invaded range [84]. Species are considered to be at equilibrium with the climate if they occur in the entire breadth of the climatically suitable areas [85]. Although niche models generally assume that the distributions of the species are at equilibrium with current climate [27], several workers have shown that the validity of this assumption varies substantially across different groups of organisms [86]. In the present study, most of the selected species are likely to be closer to equilibrium. Although empirical data for supporting this is not available, likelihood of the IAS attaining equilibrium with the climate can be argued based on the following evidences:

1. Minimum residence time: Minimum residence time of a species is the time since its first arrival in the invaded range. A longer residence period would give sufficient time for colonization and spread, and reach equilibrium with climate. It is usually ascertained through: (a) published literature, and (b) the date of the first herbarium specimen collected for the species [87]. Although data on the exact time of introduction for IAS is scarce, there is a strong correlation between known time of introduction and the date of the first herbarium collection [88]. In the present study, the residence time of 86 species was determined as more than 150 years based on the published literature and herbarium records [89–94]. Their introduction to India can be linked with the historical trade relationship with Britain, Portugal, Spain, France, Middle East and Central Asia [17]. Irrespective of the residence time, IAS do vary greatly in the rate of their spread based on species-specific traits. For instance, at least two species out of 155 viz., *Alternanthera philoxeroides* and *Mikania micrantha* which were introduced to India only in 1940s, have become highly invasive and have spread throughout the country [4, 95].
2. Species traits: Life-form and life-cycle duration e.g., annuals, which reproduce each year will have more generations than trees, even if they have arrived at the same time. At least 79 IAS out of 155 are annual herbs with r-strategic life-history having ability to produce large number of propagules, with short life span and with multiple generations within a limited time period. The extent of occurrence of each of the modelled species has a wide native range, thus having high invasive potential [15, 96].
3. Mode and rate of dispersal: Mode and rate of dispersal of the propagules determine how fast the IAS would reach and colonize new areas [97]. Inclusion of proxy variables representing various processes that contribute to species spread viz., network of roads has been shown to enhance model accuracy [98–100]. The high density of road network and human habitations in India is likely to facilitate localized dispersal of IAS in the vicinity of occupied sites, besides contributing towards high propagule pressure. The road network and human habitations also enhance the invasion potential by creating disturbance gradients at landscape level [101]. Further, the spatial extent and rate of dispersal of propagules of selected IAS are likely to be augmented by biotic agents, the predominant mode of dispersal, especially the highly mobile human beings [102].

Thus, great dispersal abilities coupled with a reasonably longer residence time of the selected suite of IAS, reflects its high likelihood of being in equilibrium with the climate.

Responses of plant species, in general, to climate change are more likely to be accurately forecasted by models correlating present day distributions with climate than the animal groups [86]. IAS benefit from a variety of global change factors including enhanced nitrogen deposition, increased atmospheric CO_2_ concentrations, habitat fragmentation and other disturbances [103–105]. In addition, as argued by Colautti and Lau [106], the contemporary evolution may provide invasive species an evolutionary advantage over native species because of: (i) better adaptation due to co-evolution of IAS and humans [107], (ii) the act of multiple introductions from different geographical sources that increase genetic variation in physical and ecological traits, and produce novel phenotypes facilitating adaptation in introduced regions [108], (iii) greater population size and high density of IAS enhances the probability of adaptive mutations against changing environment ensuring competitive advantage over natives [109], and (iv) invaders escape many evolutionary constraints by leaving behind their specialized natural enemies in the native range [110], providing selection opportunity on multiple traits and more freedom to adapt in comparison to the native species.

In the present study, the ExDet analysis shows certain areas in the Western Ghats, Western Himalaya, and northeastern region of India as novel in some climatic aspects (S1 and S2 Figs). The predicted climatic suitability for IAS in these regions, and their presence as validated through field surveys indicates the robustness of the predicted hotspots. However, to have further explicit and mechanistic explanations, a fine grain empirical analysis at ecosystem, provenance and intraspecific level is required [111].

The above evidence suggests of possibilities, where species evolve, often rapidly under selection, leading to differences in population traits from original introduction [112, 113]. Further, local adaptations also enable the IAS to invade novel regions [111] resulting in the expansion of distributional range. These processes are over and above the likelihood of being in equilibrium with current climatic conditions, and thus would have little bearing on the current study. The availability of empirical data pertaining to these variables and processes in future would further enhance model accuracy and predictions.

## Supporting Information

S1 Fig Extrapolation detection (ExDet) analysis showing areas with two types of novel conditions in the Indian subcontinental region against the model training regions: Africa (A), Australia (B), Europe (C), North America (D), and South America (E). Regions with red color have 'Type 1' novelty, and blue color have 'Type 2' novelty. Regions in green color represents similar environmental conditions. The 'Type 1' novelty represent points that are outside the range of individual covariates, and 'Type 2' novelty represent the points that are within the individual covariate range but have novel combinations between the covariates.
(TIF)

S2 Fig Extrapolation detection (ExDet) output map depicting the distribution of most influential covariate (MIC) out of the 19 bioclimatic variables which contributed to the 'Type 1' and 'Type 2' novelty in the Indian subcontinental region against the model training regions of Africa (Fig A), Australia (Fig B), Europe (Fig C), North America (Fig D), and South America (Fig E). Individual colours along with numbers below the colour ramp depict the MIC i.e. the bioclimatic variables. Blue areas in the map, labelled as 'none' in the colour legend, do not have any covariate which falls outside the range of native range bioclimatic data or have any non-analogous combination of these covariates.
(TIF)

S3 Fig Proportion of area under different consensus classes in each biome types of India. (TIF)

S4 Fig Proportion of area under different consensus classes in each of the ecoregions of India. (TIF)

S1 Table Table showing the results of continent-wise species-specific model calibration tests. (DOCX)

S2 Table Distribution of model consensus classes in the ecoregions of India. The values in the table represent the number of pixels (size: ≈ 4 km) under each class.
(DOCX)

## References

[1] Sanders NJ, Gotelli NJ, Heller NE, Gordon DM (2003) Community disassembly by an invasive species. Proc Natl Acad Sci U S A 100: 2474–2477. 12604772 10.1073/pnas.0437913100 PMC151365

[2] Lin W, Zhou G, Cheng X, Xu R (2007) Fast economic development accelerates biological invasions in China. PLoS One 2: e1208 18030342 10.1371/journal.pone.0001208 PMC2065902

[3] Thuiller W, Richardson DM, Midgley GF Will climate change promote alien plant invasions? In: Nentwig W, editor. Biological invasions. Berlin Heidelberg: Springer; 2007 pp. 197–211.

[4] Bhatt J, Singh J, Singh S, Tripathi R, Kohli R. Invasive Alien Plants An Ecological Appraisal for the Indian Subcontinent. Vol. 1 Wallingford, UK: CABI; 2011.

[5] Kelly AE, Goulden ML (2008) Rapid shifts in plant distribution with recent climate change. Proc Natl Acad Sci U S A 105: 11823–11826. 10.1073/pnas.0802891105 18697941 PMC2575286

[6] Walther GR, Roques A, Hulme PE, Sykes MT, Pyšek P, Kühn I, et al (2009) Alien species in a warmer world: risks and opportunities. Trends Ecol Evol 24: 686–693. 10.1016/j.tree.2009.06.008 19712994

[7] Gopinathan M, Babu C (1982) Cytogenetics of *Galinsoga parviflora* Cav. and *G*. *ciliata* (Raf.) Blake, and their natural hybrids (Asteraceae). New Phytol 531–539.

[8] McNeely JA. The great reshuffling: human dimensions of invasive alien species Gland, Switzerland and Cambridge, UK: IUCN; 2001.

[9] Annapurna C, Singh J (2003) Variation of *Parthenium hysterophorus* in response to soil quality: implications for invasiveness. Weed Res 43: 190–198.

[10] Raghubanshi AS RL, Gaur JP, Singh JS (2005) Invasive alien species and biodiversity in India. Curr Sci 88: 539–540.

[11] Negi PS, Hajra PK (2007) Alien flora of Doon Valley, Northwest Himalaya. Curr Sci 92: 968–978.

[12] Khuroo AA, Rashid I, Reshi Z, Dar G, Wafai B (2007) The alien flora of Kashmir Himalaya. Biol Invasions 9: 269–292.

[13] Reddy CS (2008) Catalogue of invasive alien flora of India. Life Sci J 5: 84–89.

[14] Reddy CS, Pattanaik C (2009) An assessment of floristic diversity of Gandhamardan hill range, Orissa, India. Bangladesh Journal of Plant Taxonomy 16: 29–36.

[15] Shah MA, Reshi ZA, Lavoie C (2011) Predicting plant invasiveness from native range size: clues from the Kashmir Himalaya. J Plant Ecol 5: 167–173.

[16] Sekar KC (2012) Invasive alien plants of Indian Himalayan region-diversity and implication. Am J Plant Sci 3: 177–184.

[17] Khuroo AA, Reshi ZA, Malik AH, Weber E, Rashid I, Dar GH (2012) Alien flora of India: taxonomic composition, invasion status and biogeographic affiliations. Biol Invasions 14: 99–113.

[18] Jaryan V, Uniyal SK, Kumar A, Gupta R, Singh R (2013) Extent of occurrence and area of occupancy of tallow tree (*Sapium sebiferum*): using the red list criteria for documenting invasive species expanse. National Academy Science Letters 36: 85–91.

[19] Peh KS-H (2010) Invasive species in Southeast Asia: the knowledge so far. Biodivers Conserv 19: 1083–1099.

[20] Barik SK, Adhikari D. Predicting the geographical distribution of an invasive species (*Chromolaena odorata* L. (King) & HE Robins) in the Indian subcontinent under climate change scenarios In: Bhatt JR, Singh JS, Tripathi RS, Kohli RK, editors. Invasive alien plants: an ecological appraisal for the Indian subcontinent, Wallingford, UK: CABI; 2011 pp. 77–88.

[21] Reshi ZA, Khuroo AA (2012) Alien Plant Invasions in India: Current Status and Management Challenges. Proc Natl Acad Sci India Sect B Biol Sci 82: 305–312.

[22] Thuiller W, Richardson DM, Pyšek P, Midgley GF, Hughes GO, Rouget M (2005) Niche‐based modelling as a tool for predicting the risk of alien plant invasions at a global scale. Glob Chang Biol 11: 2234–2250. 10.1111/j.1365-2486.2005.001018.x 34991288

[23] Myers N, Mittermeier RA, Mittermeier CG, Da Fonseca GA, Kent J (2000) Biodiversity hotspots for conservation priorities. Nature 403: 853–858. 10706275 10.1038/35002501

[24] O'Donnell J, Gallagher RV, Wilson PD, Downey PO, Hughes L, Leishman MR (2012) Invasion hotspots for non‐native plants in Australia under current and future climates. Glob Chang Biol 18: 617–629.

[25] Adhikari D, Barik S, Upadhaya K (2012) Habitat distribution modelling for reintroduction of *Ilex khasiana* Purk., a critically endangered tree species of northeastern India. Ecol Eng 40: 37–43.

[26] Yang X-Q, Kushwaha S, Saran S, Xu J, Roy P (2013) Maxent modeling for predicting the potential distribution of medicinal plant, *Justicia adhatoda* L. in Lesser Himalayan foothills. Ecol Eng 51: 83–87.

[27] Pearson RG, Dawson TP (2003) Predicting the impacts of climate change on the distribution of species: are bioclimate envelope models useful? Glob Ecol Biogeogr 12: 361–371.

[28] Elith J, Graham CH, P Anderson R, Dudík M, Ferrier S, Guisan A, et al (2006) Novel methods improve prediction of species’ distributions from occurrence data. Ecography 29: 129–151.

[29] Woodward FI. Climate and plant distribution Cambridge University Press 1987.

[30] Willis KJ, Whittaker RJ (2002) Species diversity-scale matters. Science 295: 1245–1248. 11847328 10.1126/science.1067335

[31] Peterson AT (2011) Ecological niche conservatism: A time‐structured review of evidence. J Biogeogr 38: 817–827.

[32] Peterson AT. Ecological niches and geographic distributions, Monographs in Population Biology; no. 49 Princeton University Press 2011.

[33] Panetta FD, Mitchell ND (1991) Homoclime analysis and the prediction of weediness. Weed Res 31: 273–284.

[34] Scott JK, Panetta FD (1993) Predicting the Australian weed status of southern African plants. J Biogeogr 20: 87–93.

[35] Pyšek P, Jarošík V, Kučera T (2003) Inclusion of native and alien species in temperate nature reserves: an historical study from Central Europe. Conserv Biol 17: 1414–1424.

[36] Curnutt JL (2000) Host-area specific climatic-matching: similarity breeds exotics. Biol Conserv 94: 341–351.

[37] Hayes KR, Barry SC (2008) Are there any consistent predictors of invasion success? Biol Invasions 10: 483–506.

[38] Schmitt CB, Burgess ND, Coad L, Belokurov A, Besançon C, Boisrobert L, et al (2009) Global analysis of the protection status of the world’s forests. Biol Conserv 142: 2122–2130.

[39] Mittermeier RA, Turner WR, Larsen FW, Brooks TM, Gascon C. Global biodiversity conservation: the critical role of hotspots In: Zachos FE, Habel JC, editors. Biodiversity hotspots. Berlin Heidelberg: Springer; 2011 pp. 3–22.

[40] Ellis EC, Ramankutty N (2008) Anthropogenic Biomes of the World, Version 1. Palisades, NY: NASA Socioeconomic Data and Applications Center (SEDAC) http://sedac.ciesin.columbia.edu/data/set/anthromes-anthropogenic-biomes-world-v1.

[41] Beaumont LJ, Gallagher RV, Thuiller W, Downey PO, Leishman MR, Hughes L (2009) Different climatic envelopes among invasive populations may lead to underestimations of current and future biological invasions. Divers Distrib 15: 409–420.

[42] Phillips SJ, Anderson RP, Schapire RE (2006) Maximum entropy modeling of species geographic distributions. Ecol Modell 190: 231–259.

[43] Olson DM, Dinerstein E, Wikramanayake ED, Burgess ND, Powell GV, Underwood EC, et al (2001) Terrestrial Ecoregions of the World: A New Map of Life on Earth A new global map of terrestrial ecoregions provides an innovative tool for conserving biodiversity. Bioscience 51: 933–938.

[44] Grace J (1987) Climatic tolerance and the distribution of plants. New Phytol 106: 113–130.

[45] Luoto M, Virkkala R, Heikkinen RK (2007) The role of land cover in bioclimatic models depends on spatial resolution. Glob Ecol Biogeogr 16: 34–42.

[46] Vicente J, Alves P, Randin C, Guisan A, Honrado J (2010) What drives invasibility? A multi‐model inference test and spatial modelling of alien plant species richness patterns in northern Portugal. Ecography 33: 1081–1092.

[47] Guisan A, Zimmermann NE (2000) Predictive habitat distribution models in ecology. Ecol modell 135: 147–186.

[48] Guisan A, Thuiller W (2005) Predicting species distribution: offering more than simple habitat models. Ecol Lett 8: 993–1009. 10.1111/j.1461-0248.2005.00792.x 34517687

[49] Guisan A, Graham CH, Elith J, Huettmann F (2007) Sensitivity of predictive species distribution models to change in grain size. Divers Distrib 13: 332–340.

[50] Elith J, Leathwick JR (2009) Species distribution models: ecological explanation and prediction across space and time. Annu Rev Ecol Evol Syst 40: 677.

[51] Hijmans RJ, Cruz JM, Rojas E, Guarino L (2001) DIVA-GIS. A geographic information system for the management and analysis of genetic resources data. Manual (Internet) Lima (Peru): International Potato Center and International Plant Genetic Resources Institute Available from: http://www.diva-gis.org.

[52] Warren DL, Glor RE, Turelli M (2010) ENMTools: a toolbox for comparative studies of environmental niche models. Ecography 33: 607–611.

[53] Pearson RG, Raxworthy CJ, Nakamura M, Townsend Peterson A (2007) Predicting species distributions from small numbers of occurrence records: a test case using cryptic geckos in Madagascar. J Biogeogr 34: 102–117.

[54] Mesgaran MB, Cousens RD, Webber BL (2014) Here be dragons: a tool for quantifying novelty due to covariate range and correlation change when projecting species distribution models. Diversity and Distributions 20: 1147–1159.

[55] Elith J, Phillips SJ, Hastie T, Dudík M, Chee YE, Yates CJ (2011) A statistical explanation of MaxEnt for ecologists. Divers Distrib 17: 43–57.

[56] Phillips SJ, Dudík M (2008) Modeling of species distributions with Maxent: new extensions and a comprehensive evaluation. Ecography 31: 161–175.

[57] Thuiller W, Broennimann O, Hughes G, Alkemade JRM, Midgley GF, Corsi F (2006) Vulnerability of African mammals to anthropogenic climate change under conservative land transformation assumptions. Glob Chang Biol 12: 424–440.

[58] Escalante T, Rodríguez-Tapia G, Linaje M, Illoldi-Rangel P, González-López R (2013) Identification of areas of endemism from species distribution models: threshold selection and Nearctic mammals. TIP Revista Especializada en Ciencias Químico-Biológicas 16: 5–17.

[59] Phillips SJ, Dudík M, Elith J, Graham CH, Lehmann A, Leathwick J, et al (2009) Sample selection bias and presence-only distribution models: implications for background and pseudo-absence data. Ecol Appl 19: 181–197. 19323182 10.1890/07-2153.1

[60] Peterson AT, Vieglais DA (2001) Predicting Species Invasions Using Ecological Niche Modeling: New Approaches from Bioinformatics Attack a Pressing Problem A new approach to ecological niche modeling, based on new tools drawn from biodiversity informatics, is applied to the challenge of predicting potential species' invasions. Bioscience 51: 363–371.

[61] Jiménez-Valverde A, Nakazawa Y, Lira-Noriega A, Peterson AT (2009) Environmental correlation structure and ecological niche model projections. Biodiversity Informatics 6: 28–35.

[62] Levine JM, D'Antonio CM (1999) Elton revisited: a review of evidence linking diversity and invasibility. Oikos 87: 15–26.

[63] Stadler J, Trefflich A, Klotz S, Brandl R (2000) Exotic plant species invade diversity hot spots: the alien flora of northwestern Kenya. Ecography 23: 169–176.

[64] Parepa M, Fischer M, Bossdorf O (2013) Environmental variability promotes plant invasion. Nat Commun 4: 1604 10.1038/ncomms2632 23511469

[65] Stohlgren TJ, Binkley D, Chong GW, Kalkhan MA, Schell LD, Bull KA, et al (1999) Exotic plant species invade hot spots of native plant diversity. Ecol Monogr 69: 25–46.

[66] India State of Forest Report (2011) Forest Survey of India, Ministry of Environment and Forests, Dehradun.

[67] Sloan S, Jenkins CN, Joppa LN, Gaveau DL, Laurance WF (2014) Remaining natural vegetation in the global biodiversity hotspots. Biol Conserv 177: 12–24.

[68] Jha CS, Dutt CBS, Bawa KS (2000) Deforestation and land use changes in Western Ghats, India. Curr Sci 79: 231–237.

[69] Tilman D, Lehman C (2001) Human-caused environmental change: impacts on plant diversity and evolution. Proc Natl Acad Sci U S A 98: 5433–5440. 11344290 10.1073/pnas.091093198 PMC33230

[70] Shea K, Chesson P (2002) Community ecology theory as a framework for biological invasions. Trends Ecol Evol 17: 170–176.

[71] Gravuer K, Sullivan JJ, Williams PA, Duncan RP (2008) Strong human association with plant invasion success for *Trifolium* introductions to New Zealand. Proc Natl Acad Sci U S A 105: 6344–6349. 10.1073/pnas.0712026105 18427111 PMC2359821

[72] Catford JA, Downes BJ, Gippel CJ, Vesk PA (2011) Flow regulation reduces native plant cover and facilitates exotic invasion in riparian wetlands. J Appl Ecol 48: 432–442.

[73] Bellard C, Leclerc C, Leroy B, Bakkenes M, Veloz S, Thuiller W, et al (2014) Vulnerability of biodiversity hotspots to global change. Glob Ecol Biogeogr 23: 1376–1386.

[74] Moles AT, Gruber MA, Bonser SP (2008) A new framework for predicting invasive plant species. J Ecol 96: 13–17.

[75] Lekevičius E (2009) Vacant niches in nature, ecology, and evolutionary theory: a mini-review. Ekologija 55: 165–174.

[76] Denslow JS (2003) Weeds in paradise: thoughts on the invasibility of tropical islands. Annals of the Missouri Botanical Garden 90: 119–127.

[77] King JR, Tschinkel WR (2008) Experimental evidence that human impacts drive fire ant invasions and ecological change. Proc Natl Acad Sci U S A 105: 20339–20343. 10.1073/pnas.0809423105 19064909 PMC2629336

[78] Roura-Pascual N, Hui C, Ikeda T, Leday G, Richardson DM, Carpintero S, et al (2011) Relative roles of climatic suitability and anthropogenic influence in determining the pattern of spread in a global invader. Proc Natl Acad Sci U S A 108: 220–225. 10.1073/pnas.1011723108 21173219 PMC3017164

[79] Lonsdale WM (1999) Global patterns of plant invasions and the concept of invasibility. Ecology 80: 1522–1536.

[80] Ali R (2006) Issues relating to invasives in the Andaman Islands. Journal-Bombay Natural History Society 103: 349.

[81] Biswas SR, Choudhury JK, Nishat A, Rahman MM (2007) Do invasive plants threaten the Sundarbans mangrove forest of Bangladesh? Forest Ecology and Management 245: 1–9.

[82] Rasingam L, Parthasarathy N (2009) Diversity of understory plants in undisturbed and disturbed tropical lowland forests of Little Andaman Island, India. Biodiversity and conservation 18: 1045–1065.

[83] Hulme PE (2007) Biological invasions in Europe: drivers, pressures, states, impacts and responses. Biodiversity under threat 25: 56–80.

[84] Peterson AT, Soberón J, Sánchez-Cordero V (1999) Conservatism of ecological niches in evolutionary time. Science 285: 1265–1267. 10455053 10.1126/science.285.5431.1265

[85] Hutchinson GE (1957) Cold Spring Harbor symposium on quantitative biology. Concluding remarks 22: 415–427.

[86] Araújo MB, Pearson RG (2005) Equilibrium of species’ distributions with climate. Ecography 28: 693–695.

[87] Gallagher RV, Beaumont LJ, Hughes L, Leishman MR (2010) Evidence for climatic niche and biome shifts between native and novel ranges in plant species introduced to Australia. J Ecol 98: 790–799.

[88] Hamilton MA, Murray BR, Cadotte MW, Hose GC, Baker AC, Harris CJ, et al (2005) Life‐history correlates of plant invasiveness at regional and continental scales. Ecol Lett 8: 1066–1074.

[89] Hooker JD. The Flora of British India. I, Ashford, Kent, England: L. Reeve & Co., Ltd; 1872.

[90] Hooker JD. The Flora of British India. II, Ashford, Kent, England: L. Reeve & Co. Ltd; 1879.

[91] Hooker JD. The Flora of British India. III, Ashford, Kent, England: L. Reeve & Co. Ltd; 1882.

[92] Hooker JD. The Flora of British India. IV, Ashford, Kent, England: L. Reeve & Co. Ltd; 1885.

[93] Hooker JD. The Flora of British India. V, Ashford, Kent, England: L. Reeve & Co. Ltd; 1885.

[94] Hooker JD. The Flora of British India. VI, Ashford, Kent, England: L. Reeve & Co. Ltd; 1894.

[95] Maheshwari JK (1965) Alligator weed in Indian lakes. Nature 206: 1270.4956348

[96] Dawson W, Rohr RP, van Kleunen M, Fischer M (2012) Alien plant species with a wider global distribution are better able to capitalize on increased resource availability. New Phytol 194: 859–867. 10.1111/j.1469-8137.2012.04104.x 22409575

[97] Wilson JR, Richardson DM, Rouget M, Procheş Ş, Amis MA, Henderson L, et al (2007) Residence time and potential range: crucial considerations in modelling plant invasions. Divers Distrib 13: 11–22.

[98] Dullinger S, Kleinbauer I, Peterseil J, Smolik M, Essl F (2009) Niche based distribution modelling of an invasive alien plant: effects of population status, propagule pressure and invasion history. Biol Invasions 11: 2401–2414.

[99] Allouche O, Steinitz O, Rotem D, Rosenfeld A, Kadmon R (2008) Incorporating distance constraints into species distribution models. J Appl Ecol 45: 599–609.

[100] Václavík T, Meentemeyer RK (2012) Equilibrium or not? Modelling potential distribution of invasive species in different stages of invasion. Divers Distrib 18: 73–83.

[101] Leu M, Hanser SE, Knick ST (2008) The human footprint in the west: a large-scale analysis of anthropogenic impacts. Ecol Appl 18: 1119–1139. 18686576 10.1890/07-0480.1

[102] Mack RN, Lonsdale WM (2001) Humans as global plant dispersers: getting more than we bargained for. Bioscience 51: 95–102.

[103] Dukes JS, Mooney HA (1999) Does global change increase the success of biological invaders? Trends Ecol Evol 14: 135–139. 10322518 10.1016/s0169-5347(98)01554-7

[104] Visser ME (2008) Keeping up with a warming world; assessing the rate of adaptation to climate change. Proc Biol Sci 275: 649–659. 10.1098/rspb.2007.0997 18211875 PMC2409451

[105] Merilä J, Hendry AP (2014) Climate change, adaptation, and phenotypic plasticity: the problem and the evidence. Evol Appl 7: 1–14. 10.1111/eva.12137 24454544 PMC3894893

[106] Colautti RI, Lau JA (2015) Contemporary evolution during invasion: evidence for differentiation, natural selection, and local adaptation. Mol Ecol 24: 1999–2017. 10.1111/mec.13162 25891044

[107] Hufbauer RA, Facon B, Ravigne V, Turgeon J, Foucaud J, Lee CE, et al (2012) Anthropogenically induced adaptation to invade (AIAI): contemporary adaptation to human‐altered habitats within the native range can promote invasions. Evol Appl 5: 89–101. 10.1111/j.1752-4571.2011.00211.x 25568032 PMC3353334

[108] Rius M, Darling JA (2014) How important is intraspecific genetic admixture to the success of colonising populations? Trends Ecol Evol 29: 233–242. 10.1016/j.tree.2014.02.003 24636862

[109] Gomulkiewicz R, Holt RD (1995) When does evolution by natural selection prevent extinction? Evolution 49: 201–207. 28593677 10.1111/j.1558-5646.1995.tb05971.x

[110] Strauss SY (2014) Ecological and evolutionary responses in complex communities: implications for invasions and eco‐evolutionary feedbacks. Oikos 123: 257–266.

[111] Zenni RD, Bailey JK, Simberloff D (2014) Rapid evolution and range expansion of an invasive plant are driven by provenance–environment interactions. Mol Ecol 17: 727–735. 10.1111/ele.12278 24703489

[112] Maron JL, Vilà M, Bommarco R, Elmendorf S, Beardsley P (2004) Rapid evolution of an invasive plant. Ecol Monogr 74: 261–280.

[113] Vandepitte K, Meyer T, Helsen K, Acker K, Roldán‐Ruiz I, Mergeay J, et al (2014) Rapid genetic adaptation precedes the spread of an exotic plant species. Mol Ecol 23: 2157–2164. 10.1111/mec.12683 24479960

