# Supplementary figures and images for: Modelling Hotspots for Invasive Alien Plants in India
Source: PLoS One. 2015 Jul 31;10(7):e0134665. doi: 10.1371/journal.pone.0134665 (PMC4521859; doi:10.1371/journal.pone.0134665)

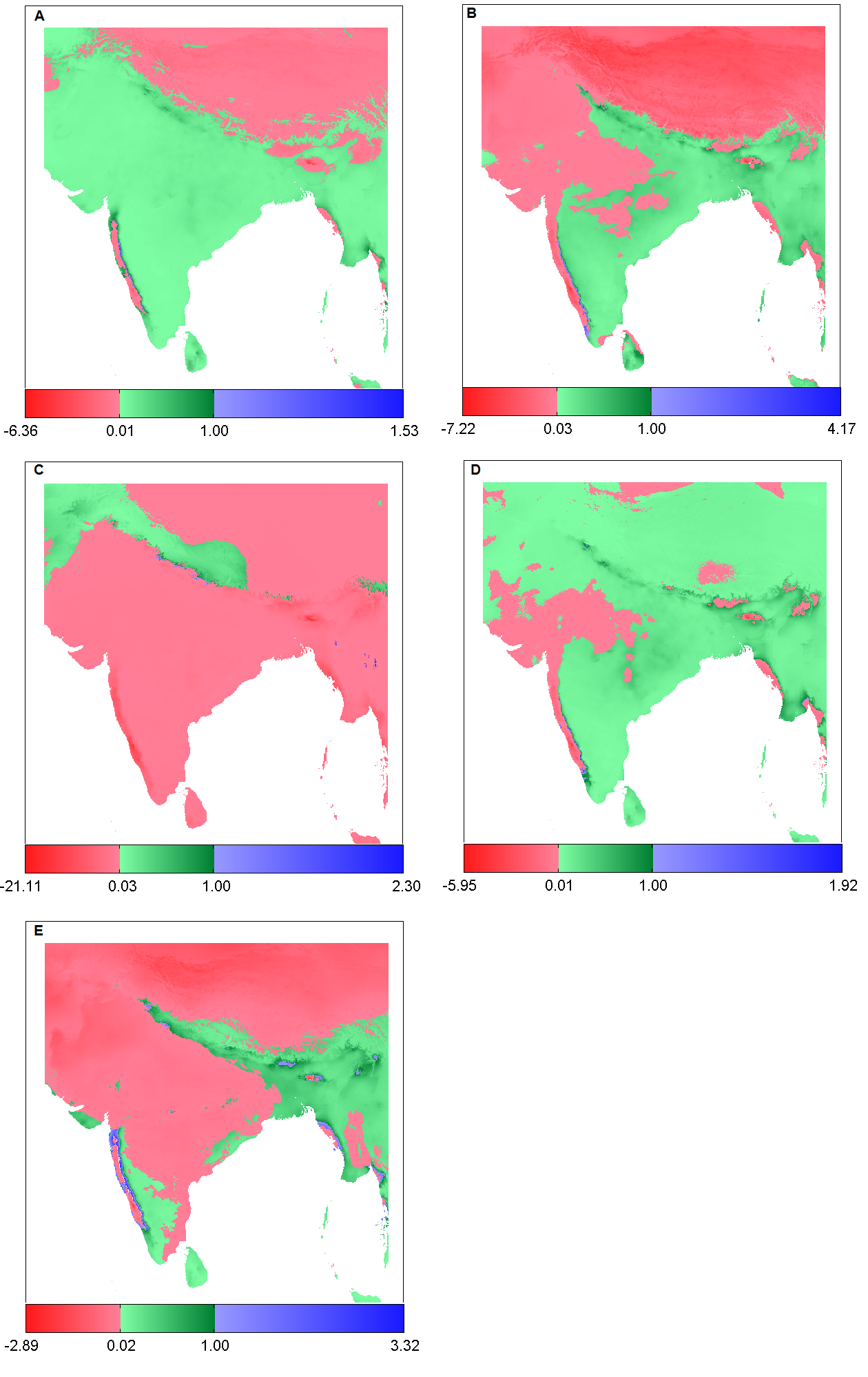

Supplement: S1 Fig — Regions with red color have 'Type 1' novelty, and blue color have 'Type 2' novelty. Regions in green color represents similar environmental conditions. The 'Type 1' novelty represent points that are outside the range of individual covariates, and 'Type 2' novelty represent the points that are within the individual covariate range but have novel combinations between the covariates. (TIF) [file pone.0134665.s001.tif]

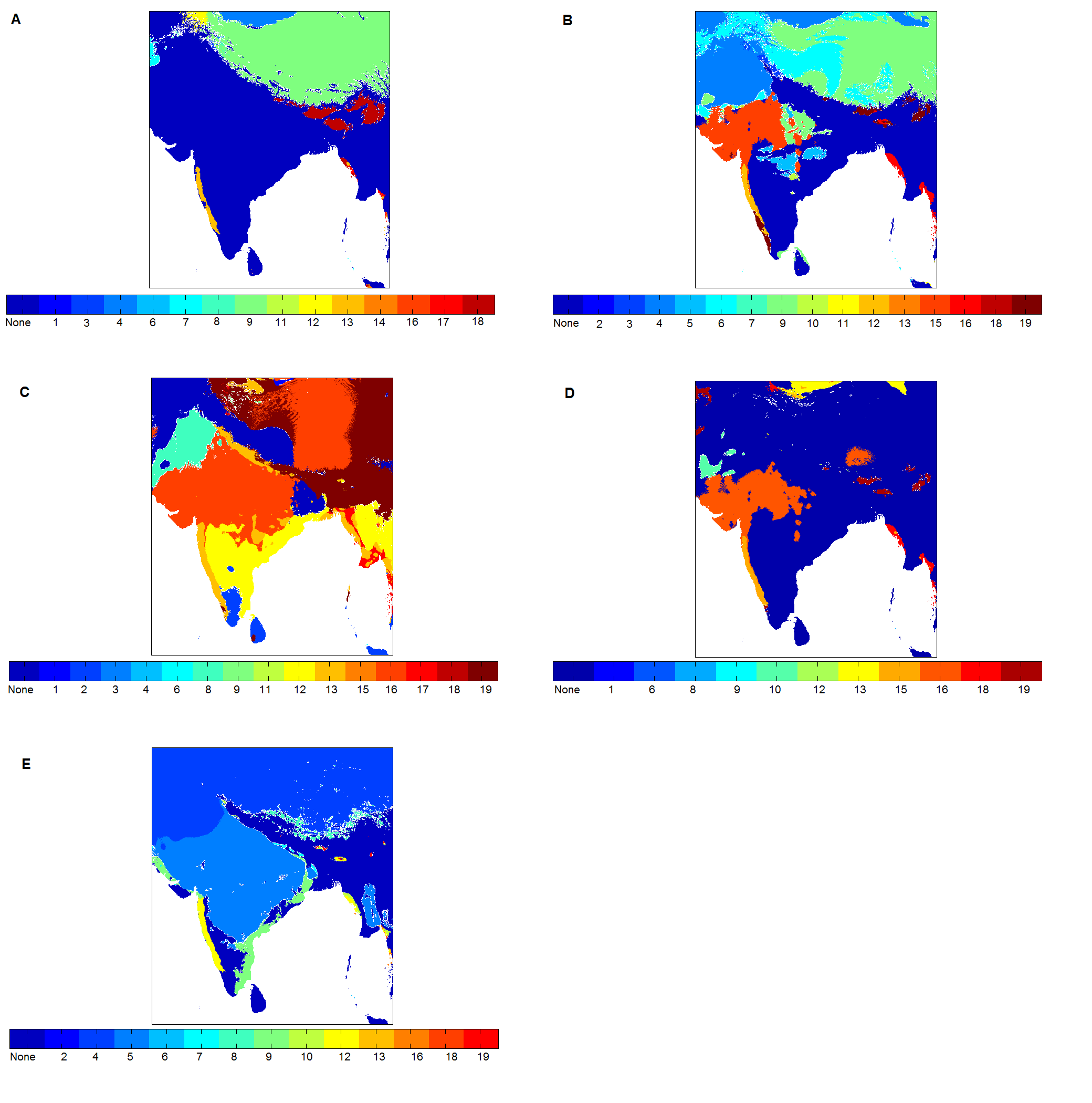

Supplement: S2 Fig — Individual colours along with numbers below the colour ramp depict the MIC i.e. the bioclimatic variables. Blue areas in the map, labelled as 'none' in the colour legend, do not have any covariate which falls outside the range of native range bioclimatic data or have any non-analogous combination of these covariates. (TIF) [file pone.0134665.s002.tif]

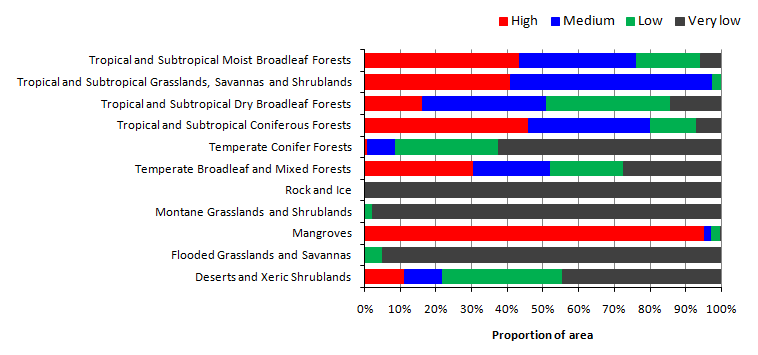

Supplement: S3 Fig — (TIF) [file pone.0134665.s003.tif]

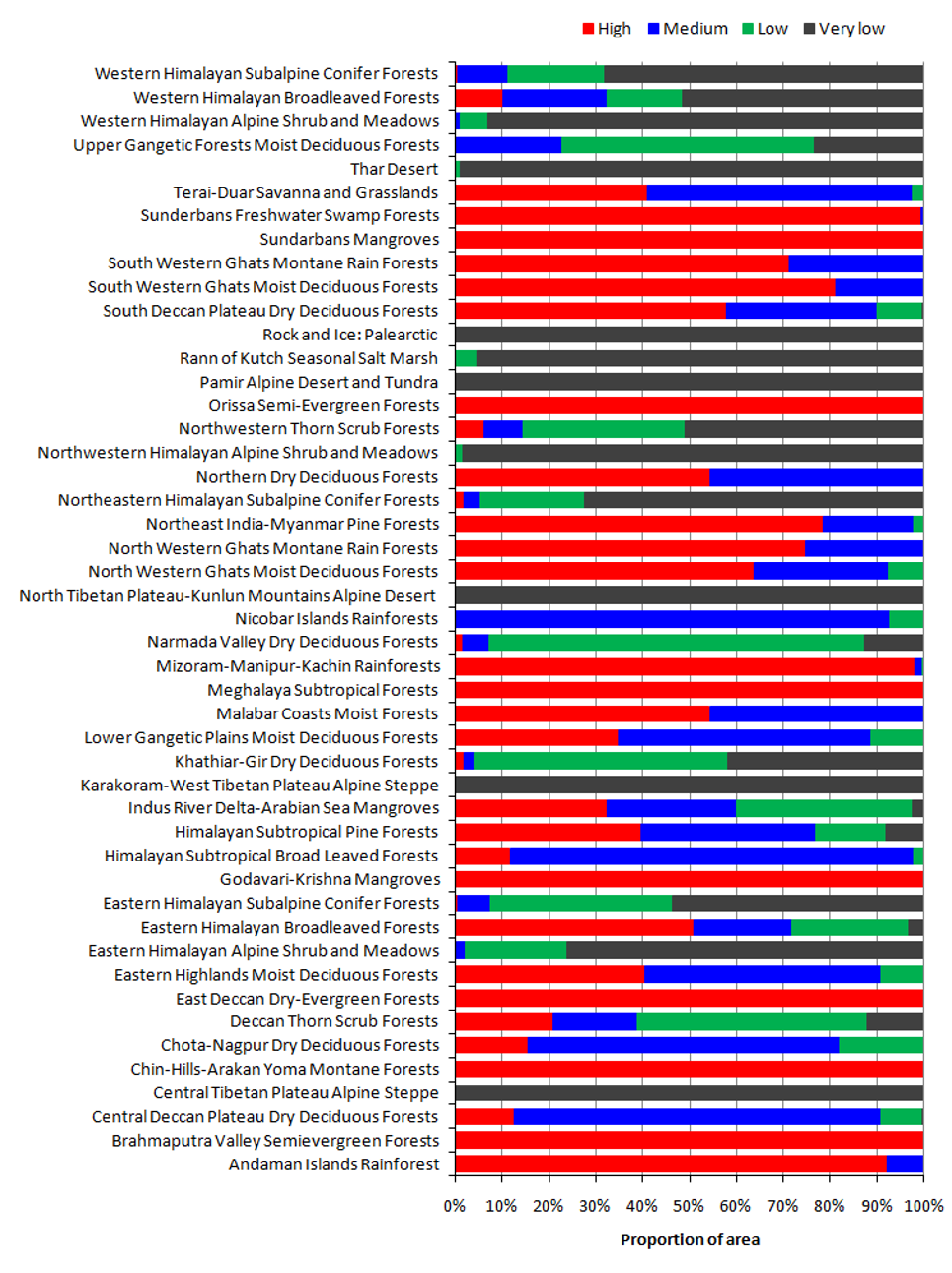

Supplement: S4 Fig — (TIF) [file pone.0134665.s004.tif]
