# Supplementary material for: Modelling Hotspots for Invasive Alien Plants in India
Source: PLoS One. 2015 Jul 31;10(7):e0134665. doi: 10.1371/journal.pone.0134665 (PMC4521859; doi:10.1371/journal.pone.0134665)
Supplement: S1 Table — (DOCX) [file pone.0134665.s005.docx]

**S1 Table. Results of continent-wise species-specific model calibration tests.**

| Continent/ Species | Family | Life form | Life cycle | Native region | Occurrence data | | | Mean AUC values | |
| --- | --- | --- | --- | --- | --- | --- | --- | --- | --- |
|  |  |  |  |  | **Train** | **Test** | **Total** | **Train** | **Test** |
| Africa |  |  |  |  |  |  |  |  |  |
| *Acacia farnesiana* | Leguminosae | Shrub | Perennial | Tropical America | 54 | 17 | 71 | 0.98 | 0.92 |
| *Acalypha ciliata* | Euphorbiacaea | Herb | Annual | Tropical Africa | 68 | 22 | 90 | 0.93 | 0.9 |
| *Acanthospermum hispidium* | Asteraceae | Herb | Annual | Tropical America | 98 | 32 | 130 | 0.94 | 0.85 |
| *Achyranthes aspera* | Amaranthaceae | Herb | Perennial | Tropical America | 323 | 107 | 430 | 0.91 | 0.93 |
| *Aerva japonica* | Amaranthaceae | Herb | Perennial | Tropical Asia | 41 | 13 | 54 | 0.92 | 0.89 |
| *Aeschynomene americana* | Leguminosae | Herb | Perennial | Tropical America | 27 | 8 | 35 | 0.98 | 0.91 |
| *Aeschynomene indica* | Leguminosae | Herb | Annual | North & Central America | 105 | 34 | 139 | 0.94 | 0.9 |
| *Ageratum conyzoides* | Asteraceae | Herb | Annual | South America | 233 | 77 | 310 | 0.93 | 0.9 |
| *Alternanthera pungens* | Amaranthaceae | Herb | Perennial | Tropical America | 108 | 36 | 144 | 0.97 | 0.98 |
| *Alternanthera sessilis* | Amaranthaceae | Herb | Perennial | Tropical America | 151 | 50 | 201 | 0.93 | 0.85 |
| *Ambrosia artemisiifolia* | Asteraceae | Shrub | Annual | North America | 18 | 6 | 24 | 0.97 | 0.99 |
| *Anthemis cotula* | Asteraceae | Herb | Annual | Temperate Eurasia | 185 | 61 | 246 | 0.9 | 0.84 |
| *Antigonon leptopus* | Polygonaceae | Climber | Perennial | South America | 13 | 4 | 17 | 0.93 | 0.93 |
| *Argemone mexicana* | Papaveraceae | Herb | Annual | West Indies | 42 | 13 | 55 | 0.94 | 0.94 |
| *Asclepias curassavica* | Asclepiadaceae | Herb | Perennial | Tropical America | 15 | 4 | 19 | 0.99 | 0.84 |
| *Asphodelus tenuifolius* | Asphodelaceae | Herb | Annual | Tropical Africa & West Asia | 126 | 41 | 167 | 0.99 | 0.96 |
| *Bacopa monnieri* | Scrophulariaceae | Herb | Perennial | Tropical America | 27 | 9 | 36 | 0.99 | 0.95 |
| *Bidens pilosa* | Asteraceae | Herb | Annual | South America | 205 | 68 | 273 | 0.92 | 0.91 |
| *Biophytum sensitivum* | Geraniaceae | Herb | Annual | Pantropical | 12 | 4 | 16 | 0.93 | 0.92 |
| *Blumea lacera* | Asteraceae | Herb | Annual | Tropical America | 5 | 1 | 6 | 0.83 | 1 |
| *Calotropis gigantea* | Asclepiadaceae | Shrub | Perennial | Tropical Africa | 5 | 1 | 6 | 0.9 | 0.98 |
| *Calotropis procera* | Asclepiadaceae | Shrub | Perennial | Tropical Africa | 45 | 15 | 60 | 0.94 | 0.92 |
| *Capsella bursa-pastoris* | Cruciferae | Herb | Biennial | Temperate Europe | 242 | 80 | 322 | 0.98 | 0.98 |
| *Cardamine hirsuta* | Cruciferae | Herb | Annual | Temperate Eurasia | 125 | 41 | 166 | 0.98 | 0.97 |
| *Cardamine trichocarpa* | Cruciferae | Herb | Annual | Temperate Eurasia | 18 | 5 | 23 | 0.98 | 0.97 |
| *Cardiospermum halicacabum* | Sapindaceae | Climber | Perennial | South America | 78 | 26 | 104 | 0.92 | 0.87 |
| *Celosia argentea* | Amaranthaceae | Herb | Annual | Tropical America | 47 | 15 | 62 | 0.95 | 0.86 |
| *Ceratophyllum demersum* | Ceratophyllaceae | Herb | Perennial | Tropical America | 80 | 26 | 106 | 0.95 | 0.89 |
| *Chamaecrista absus* | Leguminosae | Herb | Annual | Pantropical | 121 | 40 | 161 | 0.95 | 0.91 |
| *Chamaecrista rotundifolia* | Leguminosae | Herb | Annual | Tropical America | 34 | 11 | 45 | 0.97 | 0.96 |
| *Chenopodium album* | Chenopodiaceae | Herb | Annual | Paleotropical | 189 | 63 | 252 | 0.98 | 0.98 |
| *Chloris barbata* | Poaceae | Grass | Annual | Tropical America & Africa | 7 | 2 | 9 | 0.94 | 0.5 |
| *Chromolaena odorata* | Asteraceae | Shrub | Perennial | Central America | 62 | 20 | 82 | 0.98 | 0.91 |
| *Chrozophora plicata* | Euphorbiacaea | Herb | Annual | Tropical Africa | 22 | 7 | 29 | 0.99 | 0.99 |
| *Cissampelos pareira* | Menispermaceae | Climber | Perennial | South America | 48 | 15 | 63 | 0.98 | 0.95 |
| *Cleome gynandra* | Capparaceae | Herb | Annual | Paleotropical | 201 | 66 | 267 | 0.91 | 0.86 |
| *Cleome monophylla* | Capparaceae | Herb | Annual | Tropical Africa | 148 | 49 | 197 | 0.96 | 0.95 |
| *Cleome rutidosperma* | Capparaceae | Herb | Annual | Tropical Africa | 48 | 15 | 63 | 0.98 | 0.98 |
| *Clidemia hirta* | Melastomataceae | Shrub | Perennial | Tropical America | 20 | 6 | 26 | 1 | 1 |
| *Convolvulus arvensis* | Convolvulaceae | Climber | Perennial | Temperate Eurasia | 241 | 80 | 321 | 0.98 | 0.97 |
| *Conyza bonariensis* | Asteraceae | Herb | Annual | West Asia | 183 | 61 | 244 | 0.97 | 0.94 |
| *Corchorus aestuans* | Tiliaceae | Herb | Perennial | Tropical America | 33 | 10 | 43 | 0.96 | 0.92 |
| *Corchorus fascicularis* | Tiliaceae | Herb | Annual | Tropical America | 12 | 3 | 15 | 0.95 | 0.95 |
| *Corchorus olitorius* | Tiliaceae | Herb | Annual | Tropical Africa | 60 | 20 | 80 | 0.93 | 0.85 |
| *Corchorus tridens* | Tiliaceae | Herb | Annual | Pantropical | 72 | 23 | 95 | 0.93 | 0.9 |
| *Coronopus didymus* | Cruciferae | Herb | Biennial | Tropical America | 55 | 18 | 73 | 0.99 | 0.96 |
| *Cotula anthemoides* | Asteraceae | Herb | Annual | Europe | 72 | 24 | 96 | 0.94 | 0.92 |
| *Crassocephalum crepidioides* | Asteraceae | Herb | Annual | Tropical Africa | 103 | 34 | 137 | 0.97 | 0.95 |
| *Cyperus difformis* | Cyperaceae | Sedge | Annual | Tropical America | 178 | 59 | 237 | 0.93 | 0.91 |
| *Cyperus iria* | Cyperaceae | Sedge | Annual | Paleotropical | 42 | 13 | 55 | 0.91 | 0.9 |
| *Cyperus rotundus* | Cyperaceae | Sedge | Annual | Paleotropical | 135 | 44 | 179 | 0.93 | 0.85 |
| *Datura inoxia* | Solanaceae | Shrub | Annual | South America | 41 | 13 | 54 | 0.93 | 0.76 |
| *Datura stramonium* | Solanaceae | Shrub | Annual | Tropical America | 123 | 41 | 164 | 0.96 | 0.94 |
| *Daucus carota* | Umbelliferae | Herb | Biennial | Temperate Eurasia | 355 | 118 | 473 | 0.98 | 0.97 |
| *Desmanthus virgatus* | Leguminosae | Shrub | Perennial | Tropical America | 18 | 6 | 24 | 0.95 | 0.9 |
| *Dicoma tomentosa* | Asteraceae | Herb | Annual | Tropical Africa | 86 | 28 | 114 | 0.97 | 0.91 |
| *Digera muricata* | Amaranthaceae | Herb | Annual | North America | 10 | 3 | 13 | 0.96 | 0.78 |
| *Echinochloa colona* | Poaceae | Grass | Annual | Paleotropical | 252 | 84 | 336 | 0.9 | 0.87 |
| *Echinochloa crus-galli* | Poaceae | Grass | Annual | Paleotropical | 129 | 42 | 171 | 0.97 | 0.95 |
| *Emilia sonchifolia* | Asteraceae | Herb | Annual | Tropical Africa | 57 | 18 | 75 | 0.97 | 0.94 |
| *Euphorbia brasiliensis* | Euphorbiacaea | Herb | Annual | South America | 24 | 8 | 32 | 0.98 | 0.93 |
| *Euphorbia cyathophora* | Euphorbiacaea | Herb | Annual | North & South America | 3 | 1 | 4 | 0.93 | 0.76 |
| *Euphorbia helioscopia* | Euphorbiacaea | Herb | Annual | West Asia | 201 | 67 | 268 | 0.99 | 0.99 |
| *Euphorbia heterophylla* | Euphorbiacaea | Herb | Annual | Mexico | 102 | 33 | 135 | 0.94 | 0.89 |
| *Euphorbia hirta* | Euphorbiacaea | Herb | Annual | Tropical America | 175 | 58 | 233 | 0.92 | 0.9 |
| *Euphorbia ovalifolia* | Euphorbiacaea | Herb | Annual | South America | 50 | 16 | 66 | 0.96 | 0.97 |
| *Euphorbia thymifolia* | Euphorbiacaea | Herb | Annual | Tropical America | 25 | 8 | 33 | 0.96 | 0.98 |
| *Evolvulus nummularius* | Convolvulaceae | Herb | Annual | West Indies | 27 | 8 | 35 | 0.95 | 0.9 |
| *Galinsoga parviflora* | Asteraceae | Herb | Annual | Tropical America | 75 | 24 | 99 | 0.96 | 0.92 |
| *Gamochaeta coarctata* | Asteraceae | Herb | Perennial | South America | 19 | 6 | 25 | 0.99 | 0.99 |
| *Gamochaeta pensylvanica* | Asteraceae | Herb | Annual | South america | 24 | 8 | 32 | 0.99 | 0.99 |
| *Gnaphalium polycaulon* | Asteraceae | Herb | Annual | West Indies | 13 | 4 | 17 | 0.91 | 0.74 |
| *Gnaphalium purpureum* | Asteraceae | Herb | Annual | Tropical America | 3 | 1 | 4 | 0.96 | 0.97 |
| *Gomphrena serrata* | Amaranthaceae | Herb | Annual | Tropical America | 105 | 34 | 139 | 0.98 | 0.94 |
| *Heliotropium indicum* | Boraginaceae | Herb | Annual | South America | 34 | 11 | 45 | 0.92 | 0.94 |
| *Hyptis suaveolens* | Lamiaceae | Herb | Annual | South America | 41 | 13 | 54 | 0.98 | 0.96 |
| *Impatiens balsamina* | Balsaminaceae | Herb | Annual | Southeast Asia | 5 | 1 | 6 | 0.94 | 0.5 |
| *Ipomoea carnea* | Convolvulaceae | Shrub | Perennial | South America | 13 | 4 | 17 | 0.95 | 0.96 |
| *Ipomoea fistulosa* | Convolvulaceae | Shrub | Perennial | South America | 12 | 4 | 16 | 0.97 | 0.99 |
| *Ipomoea hederifolia* | Convolvulaceae | Climber | Annual | Tropical America | 17 | 5 | 22 | 0.99 | 0.95 |
| *Ipomoea indica* | Convolvulaceae | Climber | Perennial | South America | 42 | 14 | 56 | 0.98 | 0.95 |
| *Ipomoea obscura* | Convolvulaceae | Climber | Perennial | Paleotropical | 210 | 70 | 280 | 0.95 | 0.93 |
| *Ipomoea pes-tigridis* | Convolvulaceae | Climber | Annual | Tropical Africa | 12 | 4 | 16 | 0.9 | 0.83 |
| *Ipomoea quamoclit* | Convolvulaceae | Climber | Annual | Tropical America | 7 | 2 | 9 | 0.91 | 0.98 |
| *Lantana camara* | Verbenaceae | Shrub | Perennial | Tropical America | 132 | 43 | 175 | 0.98 | 0.94 |
| *Leonotis nepetifolia* | Lamiaceae | Shrub | Annual | Tropical America | 60 | 20 | 80 | 0.94 | 0.92 |
| *Macroptilium atropurpureum* | Leguminosae | Climber | Perennial | Tropical Central America | 15 | 5 | 20 | 0.96 | 0.91 |
| *Malvastrum coromandelianum* | Malvaceae | Herb | Annual | West Indies | 36 | 11 | 47 | 0.99 | 0.97 |
| *Martynia annua* | Martyniaceae | Herb | Annual | Tropical America | 4 | 1 | 5 | 0.96 | 0.93 |
| *Mecardonia procumbens* | Scrophulariaceae | Herb | Perennial | Tropical America | 4 | 1 | 5 | 1 | 1 |
| *Merremia aegyptia* | Convolvulaceae | Climber | Perennial | Pantropical | 24 | 8 | 32 | 0.97 | 0.96 |
| *Mimosa pudica* | Leguminosae | Herb | Perennial | South America | 54 | 17 | 71 | 0.97 | 0.97 |
| *Mirabilis jalapa* | Nyctaginaceae | Herb | Perennial | Tropical America | 21 | 7 | 28 | 0.97 | 0.87 |
| *Nicandra physalodes* | Loganiaceae | Herb | Annual | Peru | 31 | 10 | 41 | 0.97 | 0.96 |
| *Opuntia dillenii* | Cactaceae | Cactus | Perennial | Mexico | 6 | 1 | 7 | 0.84 | 0.41 |
| *Oxalis corymbosa* | Geraniaceae | Herb | Perennial | South America | 4 | 1 | 5 | 0.96 | 0.98 |
| *Oxalis pes-caprae* | Geraniaceae | Herb | Perennial | South Africa | 159 | 52 | 211 | 0.99 | 0.99 |
| *Pedalium murex* | Pedaliceae | Shrub | Annual | Tropical Africa | 12 | 3 | 15 | 0.98 | 0.98 |
| *Pennisetum purpureum* | Poaceae | Grass | Perennial | Tropical Africa | 87 | 29 | 116 | 0.96 | 0.87 |
| *Phragmites australis* | Poaceae | Grass | Perennial | Temperate Eurasia | 376 | 125 | 501 | 0.96 | 0.94 |
| *Physalis minima* | Solanaceae | Herb | Annual | Pantropical | 15 | 4 | 19 | 0.97 | 0.88 |
| *Pilea microphylla* | Urticaceae | Herb | Perennial | Tropical America | 4 | 1 | 5 | 0.83 | 0.99 |
| *Pistia stratiotes* | Araceae | Herb | Perennial | North America | 54 | 18 | 72 | 0.94 | 0.89 |
| *Polypogon monspeliensis* | Poaceae | Grass | Annual | Temperate Eurasia | 225 | 74 | 299 | 0.97 | 0.97 |
| *Portulaca quadrifida* | Portulacaceae | Herb | Annual | Pantropical | 104 | 34 | 138 | 0.96 | 0.93 |
| *Potamogeton crispus* | Haloragaceae | Herb | Perennial | Temperate Europe | 36 | 12 | 48 | 0.98 | 0.99 |
| *Prosopis juliflora* | Leguminosae | Tree | Perennial | Mexico & Central America | 4 | 1 | 5 | 0.76 | 0.99 |
| *Ricinus communis* | Euphorbiacaea | Tree | Perennial | Tropical Africa | 178 | 59 | 237 | 0.95 | 0.9 |
| *Salvinia molesta* | Salviniaceae | Herb | Perennial | South America | 23 | 7 | 30 | 0.99 | 0.84 |
| *Scoparia dulcis* | Scrophulariaceae | Herb | Annual | South America | 86 | 28 | 114 | 0.97 | 0.93 |
| *Senna alata* | Leguminosae | Shrub | Perennial | South America | 53 | 17 | 70 | 0.97 | 0.96 |
| *Senna hirsuta* | Leguminosae | Herb | Perennial | Tropical America | 49 | 16 | 65 | 0.95 | 0.95 |
| *Senna obtusifolia* | Leguminosae | Shrub | Annual | Tropical America | 93 | 30 | 123 | 0.94 | 0.84 |
| *Senna occidentalis* | Leguminosae | Shrub | Perennial | South America | 171 | 57 | 228 | 0.93 | 0.9 |
| *Sida acuta* | Malvaceae | Shrub | Perennial | Pantropical | 105 | 34 | 139 | 0.95 | 0.94 |
| *Sida cordata* | Malvaceae | Herb | Perennial | Tropical America | 8 | 2 | 10 | 0.95 | 0.99 |
| *Solanum americanum* | Solanaceae | Herb | Perennial | North & South America | 91 | 30 | 121 | 0.97 | 0.9 |
| *Solanum seaforthianum* | Solanaceae | Climber | Perennial | Tropical America | 27 | 9 | 36 | 0.98 | 0.97 |
| *Solanum torvum* | Solanaceae | Shrub | Perennial | Tropical America | 100 | 33 | 133 | 0.98 | 0.97 |
| *Spermacoce latifolia* | Rubiaceae | Herb | Perennial | South America | 26 | 8 | 34 | 0.99 | 0.97 |
| *Spermacoce scabra* | Rubiaceae | Herb | Annual | Tropical America | 3 | 1 | 4 | 0.98 | 0.6 |
| *Stachytarpheta jamaicensis* | Verbenaceae | Shrub | Perennial | South America | 42 | 13 | 55 | 0.97 | 0.92 |
| *Stachytarpheta urticifolia* | Verbenaceae | Shrub | Perennial | Tropical America | 48 | 15 | 63 | 0.97 | 0.97 |
| *Synedrella nodiflora* | Asteraceae | Herb | Annual | Tropical America | 68 | 22 | 90 | 0.96 | 0.9 |
| *Tribulus terrestris* | Zygophyllaceae | Herb | Perennial | Pantropical | 308 | 102 | 410 | 0.93 | 0.89 |
| *Tridax procumbens* | Asteraceae | Herb | Perennial | Mexico | 81 | 27 | 108 | 0.95 | 0.87 |
| *Triumfetta rhomboidea* | Tiliaceae | Shrub | Perennial | Tropical America | 170 | 56 | 226 | 0.95 | 0.92 |
| *Urena lobata* | Malvaceae | Herb | Perennial | Tropical Africa | 163 | 54 | 217 | 0.95 | 0.93 |
| *Urochloa panicoides* | Poaceae | Grass | Annual | Tropical Africa | 160 | 53 | 213 | 0.97 | 0.96 |
| *Waltheria indica* | Sterculiaceae | Shrub | Perennial | Tropical America | 219 | 72 | 291 | 0.94 | 0.92 |
| *Xanthium strumarium* | Asteraceae | Herb | Annual | South America | 104 | 34 | 138 | 0.98 | 0.97 |
| *Youngia japonica* | Asteraceae | Herb | Biennial | South America & Australia | 4 | 1 | 5 | 0.99 | 0.95 |
| Australia |  |  |  |  |  |  |  |  |  |
| *Acacia farnesiana* | Leguminosae | Shrub | Perennial | Tropical America | 297 | 127 | 424 | 0.87 | 0.8 |
| *Acanthospermum hispidium* | Asteraceae | Herb | Annual | Tropical America | 82 | 34 | 116 | 0.94 | 0.88 |
| *Achyranthes aspera* | Amaranthaceae | Herb | Perennial | Tropical America | 383 | 163 | 546 | 0.92 | 0.9 |
| *Adenostemma lavenia* | Caryophyllaceae | Herb | Perennial | South America | 70 | 30 | 100 | 0.98 | 0.97 |
| *Aerva japonica* | Amaranthaceae | Herb | Perennial | Tropical Asia | 157 | 67 | 224 | 0.96 | 0.95 |
| *Aeschynomene americana* | Leguminosae | Herb | Perennial | Tropical America | 26 | 10 | 36 | 0.98 | 0.99 |
| *Aeschynomene indica* | Leguminosae | Herb | Annual | North & Central America | 373 | 159 | 532 | 0.89 | 0.88 |
| *Ageratum conyzoides* | Asteraceae | Herb | Annual | South America | 56 | 24 | 80 | 0.97 | 0.95 |
| *Alternanthera philoxeroides* | Amaranthaceae | Herb | Perennial | South America | 52 | 22 | 74 | 0.99 | 1 |
| *Alternanthera pungens* | Amaranthaceae | Herb | Perennial | Tropical America | 168 | 71 | 239 | 0.92 | 0.87 |
| *Alternanthera sessilis* | Amaranthaceae | Herb | Perennial | Tropical America | 14 | 5 | 19 | 0.92 | 0.91 |
| *Alternanthera tenella* | Amaranthaceae | Herb | Perennial | South America | 14 | 5 | 19 | 0.98 | 0.93 |
| *Ambrosia artemisiifolia* | Asteraceae | Shrub | Annual | North America | 100 | 42 | 142 | 0.99 | 0.99 |
| *Anthemis cotula* | Asteraceae | Herb | Annual | Temperate Eurasia | 84 | 35 | 119 | 0.96 | 0.94 |
| *Antigonon leptopus* | Polygonaceae | Climber | Perennial | South America | 17 | 6 | 23 | 0.98 | 0.94 |
| *Argemone mexicana* | Papaveraceae | Herb | Annual | West Indies | 4 | 1 | 5 | 0.85 | 0.94 |
| *Asclepias curassavica* | Asclepiadaceae | Herb | Perennial | Tropical America | 85 | 36 | 121 | 0.99 | 0.93 |
| *Bacopa monnieri* | Scrophulariaceae | Herb | Perennial | Tropical America | 40 | 17 | 57 | 0.99 | 0.99 |
| *Bidens pilosa* | Asteraceae | Herb | Annual | South America | 670 | 286 | 956 | 0.96 | 0.96 |
| *Blumea lacera* | Asteraceae | Herb | Annual | Tropical America | 8 | 3 | 11 | 0.99 | 0.99 |
| *Calotropis gigantea* | Asclepiadaceae | Shrub | Perennial | Tropical Africa | 5 | 2 | 7 | 0.96 | 0.83 |
| *Calotropis procera* | Asclepiadaceae | Shrub | Perennial | Tropical Africa | 29 | 12 | 41 | 0.94 | 0.88 |
| *Capsella bursa-pastoris* | Cruciferae | Herb | Biennial | Temperate Europe | 247 | 105 | 352 | 0.96 | 0.94 |
| *Cardamine hirsuta* | Cruciferae | Herb | Annual | Temperate Eurasia | 63 | 26 | 89 | 0.98 | 0.96 |
| *Cardiospermum halicacabum* | Sapindaceae | Climber | Perennial | South America | 55 | 23 | 78 | 0.95 | 0.91 |
| *Celosia argentea* | Amaranthaceae | Herb | Annual | Tropical America | 12 | 5 | 17 | 0.99 | 0.67 |
| *Ceratophyllum demersum* | Ceratophyllaceae | Herb | Perennial | Tropical America | 45 | 19 | 64 | 0.96 | 0.92 |
| *Chamaecrista absus* | Leguminosae | Herb | Annual | Pantropical | 35 | 15 | 50 | 0.95 | 0.9 |
| *Chamaecrista rotundifolia* | Leguminosae | Herb | Annual | Tropical America | 14 | 5 | 19 | 0.98 | 0.92 |
| *Chenopodium album* | Chenopodiaceae | Herb | Annual | Paleotropical | 223 | 95 | 318 | 0.96 | 0.95 |
| *Chloris barbata* | Poaceae | Grass | Annual | Tropical America & Africa | 72 | 30 | 102 | 0.93 | 0.87 |
| *Chromolaena odorata* | Asteraceae | Shrub | Perennial | Central America | 3 | 1 | 4 | 1 | 1 |
| *Cissampelos pareira* | Menispermaceae | Climber | Perennial | South America | 5 | 2 | 7 | 1 | 1 |
| *Cleome gynandra* | Capparaceae | Herb | Annual | Paleotropical | 3 | 1 | 4 | 0.94 | 0.99 |
| *Convolvulus arvensis* | Convolvulaceae | Climber | Perennial | Temperate Eurasia | 46 | 19 | 65 | 0.97 | 0.98 |
| *Conyza bonariensis* | Asteraceae | Herb | Annual | West Asia | 690 | 295 | 985 | 0.94 | 0.93 |
| *Corchorus aestuans* | Tiliaceae | Herb | Perennial | Tropical America | 44 | 18 | 62 | 0.98 | 0.98 |
| *Corchorus fascicularis* | Tiliaceae | Herb | Annual | Tropical America | 6 | 2 | 8 | 0.99 | 0.99 |
| *Corchorus olitorius* | Tiliaceae | Herb | Annual | Tropical Africa | 14 | 5 | 19 | 0.96 | 0.73 |
| *Corchorus tridens* | Tiliaceae | Herb | Annual | Pantropical | 34 | 14 | 48 | 0.98 | 0.97 |
| *Coronopus didymus* | Cruciferae | Herb | Biennial | Tropical America | 68 | 28 | 96 | 0.94 | 0.85 |
| *Crassocephalum crepidioides* | Asteraceae | Herb | Annual | Tropical Africa | 87 | 37 | 124 | 0.99 | 0.98 |
| *Cyclospermum leptophyllum* | Umbelliferae | Herb | Annual | Tropical America | 227 | 97 | 324 | 0.98 | 0.97 |
| *Cyperus difformis* | Cyperaceae | Sedge | Annual | Tropical America | 334 | 142 | 476 | 0.89 | 0.83 |
| *Cyperus iria* | Cyperaceae | Sedge | Annual | Paleotropical | 126 | 54 | 180 | 0.88 | 0.79 |
| *Cyperus rotundus* | Cyperaceae | Sedge | Annual | Paleotropical | 252 | 108 | 360 | 0.89 | 0.85 |
| *Datura inoxia* | Solanaceae | Shrub | Annual | South America | 52 | 22 | 74 | 0.93 | 0.92 |
| *Datura stramonium* | Solanaceae | Shrub | Annual | Tropical America | 98 | 41 | 139 | 0.98 | 0.96 |
| *Daucus carota* | Umbelliferae | Herb | Biennial | Temperate Eurasia | 60 | 25 | 85 | 0.98 | 0.98 |
| *Desmanthus virgatus* | Leguminosae | Shrub | Perennial | Tropical America | 5 | 2 | 7 | 0.8 | 0.98 |
| *Dysphania ambrosioides* | Caryophyllaceae | Herb | Perennial | Tropical America | 12 | 5 | 17 | 0.98 | 0.99 |
| *Echinochloa colona* | Poaceae | Grass | Annual | Paleotropical | 248 | 106 | 354 | 0.89 | 0.86 |
| *Echinochloa crus-galli* | Poaceae | Grass | Annual | Paleotropical | 266 | 114 | 380 | 0.96 | 0.92 |
| *Emilia sonchifolia* | Asteraceae | Herb | Annual | Tropical Africa | 39 | 16 | 55 | 0.98 | 0.96 |
| *Euphorbia brasiliensis* | Euphorbiacaea | Herb | Annual | South America | 30 | 12 | 42 | 0.96 | 0.95 |
| *Euphorbia cyathophora* | Euphorbiacaea | Herb | Annual | North & South America | 21 | 9 | 30 | 0.97 | 0.98 |
| *Euphorbia helioscopia* | Euphorbiacaea | Herb | Annual | West Asia | 43 | 18 | 61 | 0.98 | 0.97 |
| *Euphorbia heterophylla* | Euphorbiacaea | Herb | Annual | Mexico | 26 | 10 | 36 | 0.96 | 0.96 |
| *Euphorbia hirta* | Euphorbiacaea | Herb | Annual | Tropical America | 106 | 45 | 151 | 0.95 | 0.94 |
| *Galinsoga parviflora* | Asteraceae | Herb | Annual | Tropical America | 80 | 34 | 114 | 0.99 | 0.98 |
| *Gamochaeta coarctata* | Asteraceae | Herb | Perennial | South America | 4 | 1 | 5 | 0.99 | 1 |
| *Gamochaeta pensylvanica* | Asteraceae | Herb | Annual | South America | 35 | 14 | 49 | 0.99 | 0.85 |
| *Gnaphalium polycaulon* | Asteraceae | Herb | Annual | West Indies | 20 | 8 | 28 | 0.85 | 0.72 |
| *Gnaphalium purpureum* | Asteraceae | Herb | Annual | Tropical America | 40 | 17 | 57 | 0.98 | 0.97 |
| *Gomphrena serrata* | Amaranthaceae | Herb | Annual | Tropical America | 138 | 59 | 197 | 0.95 | 0.9 |
| *Heliotropium indicum* | Boraginaceae | Herb | Annual | South America | 19 | 8 | 27 | 0.98 | 0.97 |
| *Hyptis suaveolens* | Lamiaceae | Herb | Annual | South America | 59 | 25 | 84 | 0.97 | 0.97 |
| *Impatiens balsamina* | Balsaminaceae | Herb | Annual | Southeast Asia | 8 | 3 | 11 | 1 | 1 |
| *Indigofera linnaei* | Leguminosae | Herb | Perennial | Tropical Africa | 163 | 69 | 232 | 0.9 | 0.78 |
| *Ipomoea carnea* | Convolvulaceae | Shrub | Perennial | South America | 6 | 2 | 8 | 0.88 | 0.61 |
| *Ipomoea hederifolia* | Convolvulaceae | Climber | Annual | Tropical America | 16 | 6 | 22 | 1 | 0.99 |
| *Ipomoea indica* | Convolvulaceae | Climber | Perennial | South America | 122 | 51 | 173 | 0.99 | 0.96 |
| *Ipomoea pes-tigridis* | Convolvulaceae | Climber | Annual | Tropical Africa | 4 | 1 | 5 | 0.99 | 0.96 |
| *Ipomoea quamoclit* | Convolvulaceae | Climber | Annual | Tropical America | 24 | 10 | 34 | 0.98 | 0.98 |
| *Lantana camara* | Verbenaceae | Shrub | Perennial | Tropical America | 739 | 316 | 1055 | 0.96 | 0.96 |
| *Leonotis nepetifolia* | Lamiaceae | Shrub | Annual | Tropical America | 5 | 1 | 6 | 0.92 | 0.87 |
| *Macroptilium atropurpureum* | Leguminosae | Climber | Perennial | Tropical Central America | 56 | 24 | 80 | 0.96 | 0.89 |
| *Malvastrum coromandelianum* | Malvaceae | Herb | Annual | West Indies | 26 | 11 | 37 | 0.98 | 0.91 |
| *Martynia annua* | Martyniaceae | Herb | Annual | Tropical America | 10 | 4 | 14 | 0.88 | 0.87 |
| *Merremia aegyptia* | Convolvulaceae | Climber | Perennial | Pantropical | 3 | 1 | 4 | 0.99 | 0.99 |
| *Mimosa pudica* | Leguminosae | Herb | Perennial | South America | 15 | 6 | 21 | 0.99 | 0.99 |
| *Mirabilis jalapa* | Nyctaginaceae | Herb | Perennial | Tropical America | 16 | 6 | 22 | 0.99 | 0.98 |
| *Monochoria vaginalis* | Pontederiaceae | Herb | Perennial | Southeast Asia | 14 | 6 | 20 | 0.99 | 0.98 |
| *Nicandra physalodes* | Loganiaceae | Herb | Annual | Peru | 33 | 14 | 47 | 0.98 | 0.99 |
| *Opuntia dillenii* | Cactaceae | Cactus | Perennial | Mexico | 5 | 1 | 6 | 0.88 | 0.6 |
| *Opuntia stricta* | Cactaceae | Cactus | Perennial | Mexico | 308 | 131 | 439 | 0.97 | 0.97 |
| *Oxalis corymbosa* | Geraniaceae | Herb | Perennial | South America | 5 | 2 | 7 | 0.95 | 0.99 |
| *Oxalis debilis* | Geraniaceae | Herb | Perennial | South America | 28 | 11 | 39 | 0.99 | 0.92 |
| *Oxalis pes-caprae* | Geraniaceae | Herb | Perennial | South Africa | 322 | 138 | 460 | 0.97 | 0.94 |
| *Pennisetum purpureum* | Poaceae | Grass | Perennial | Tropical Africa | 26 | 11 | 37 | 0.98 | 0.98 |
| *Phragmites australis* | Poaceae | Grass | Perennial | Temperate Eurasia | 462 | 198 | 660 | 0.95 | 0.94 |
| *Physalis minima* | Solanaceae | Herb | Annual | Pantropical | 53 | 22 | 75 | 0.95 | 0.94 |
| *Pilea microphylla* | Urticaceae | Herb | Perennial | Tropical America | 5 | 2 | 7 | 1 | 0.99 |
| *Pistia stratiotes* | Araceae | Herb | Perennial | North America | 14 | 6 | 20 | 0.93 | 0.93 |
| *Polypogon monspeliensis* | Poaceae | Grass | Annual | Temperate Eurasia | 289 | 123 | 412 | 0.93 | 0.91 |
| *Potamogeton crispus* | Haloragaceae | Herb | Perennial | Temperate Europe | 91 | 39 | 130 | 0.95 | 0.92 |
| *Prosopis juliflora* | Leguminosae | Tree | Perennial | Mexico & Central America | 14 | 5 | 19 | 0.9 | 0.76 |
| *Ricinus communis* | Euphorbiacaea | Tree | Perennial | Tropical Africa | 180 | 76 | 256 | 0.97 | 0.92 |
| *Ruellia tuberosa* | Acanthaceae | Herb | Perennial | Tropical America | 9 | 3 | 12 | 0.98 | 0.99 |
| *Salvinia molesta* | Salviniaceae | Herb | Perennial | South America | 53 | 22 | 75 | 0.98 | 0.93 |
| *Scoparia dulcis* | Scrophulariaceae | Herb | Annual | South America | 26 | 11 | 37 | 0.98 | 0.98 |
| *Senna alata* | Leguminosae | Shrub | Perennial | South America | 8 | 3 | 11 | 0.99 | 0.96 |
| *Senna obtusifolia* | Leguminosae | Shrub | Annual | Tropical America | 20 | 8 | 28 | 0.99 | 0.97 |
| *Senna occidentalis* | Leguminosae | Shrub | Perennial | South America | 37 | 15 | 52 | 0.94 | 0.92 |
| *Sida acuta* | Malvaceae | Shrub | Perennial | Pantropical | 52 | 22 | 74 | 0.96 | 0.91 |
| *Solanum americanum* | Solanaceae | Herb | Perennial | North & South America | 173 | 74 | 247 | 0.97 | 0.97 |
| *Solanum seaforthianum* | Solanaceae | Climber | Perennial | Tropical America | 64 | 27 | 91 | 0.99 | 0.98 |
| *Solanum torvum* | Solanaceae | Shrub | Perennial | Tropical America | 31 | 13 | 44 | 0.99 | 0.97 |
| *Spermacoce latifolia* | Rubiaceae | Herb | Perennial | South America | 7 | 2 | 9 | 0.97 | 0.99 |
| *Spermacoce scabra* | Rubiaceae | Herb | Annual | Tropical America | 5 | 1 | 6 | 1 | 1 |
| *Stachytarpheta jamaicensis* | Verbenaceae | Shrub | Perennial | South America | 17 | 6 | 23 | 0.98 | 0.92 |
| *Stachytarpheta urticifolia* | Verbenaceae | Shrub | Perennial | Tropical America | 18 | 7 | 25 | 0.99 | 0.97 |
| *Synedrella nodiflora* | Asteraceae | Herb | Annual | Tropical America | 10 | 4 | 14 | 0.99 | 1 |
| *Tribulus terrestris* | Zygophyllaceae | Herb | Perennial | Pantropical | 401 | 171 | 572 | 0.85 | 0.81 |
| *Tridax procumbens* | Asteraceae | Herb | Perennial | Mexico | 39 | 16 | 55 | 0.96 | 0.95 |
| *Triumfetta rhomboidea* | Tiliaceae | Shrub | Perennial | Tropical America | 24 | 10 | 34 | 0.99 | 0.97 |
| *Urena lobata* | Malvaceae | Herb | Perennial | Tropical Africa | 33 | 13 | 46 | 0.98 | 0.96 |
| *Urochloa panicoides* | Poaceae | Grass | Annual | Tropical Africa | 78 | 33 | 111 | 0.96 | 0.94 |
| *Waltheria indica* | Sterculiaceae | Shrub | Perennial | Tropical America | 180 | 76 | 256 | 0.94 | 0.89 |
| *Xanthium strumarium* | Asteraceae | Herb | Annual | South America | 217 | 92 | 309 | 0.95 | 0.94 |
| *Youngia japonica* | Asteraceae | Herb | Biennial | South America & Australia | 34 | 14 | 48 | 0.99 | 0.99 |
| Europe |  |  |  |  |  |  |  |  |  |
| *Acacia farnesiana* | Leguminosae | Shrub | Perennial | Tropical America | 50 | 16 | 66 | 0.99 | 1 |
| *Acanthospermum hispidium* | Asteraceae | Herb | Annual | Tropical America | 5 | 1 | 6 | 0.98 | 1 |
| *Achyranthes aspera* | Amaranthaceae | Herb | Perennial | Tropical America | 6 | 1 | 7 | 0.98 | 1 |
| *Aeschynomene indica* | Leguminosae | Herb | Annual | North & Central America | 7 | 2 | 9 | 0.96 | 0.96 |
| *Ageratum conyzoides* | Asteraceae | Herb | Annual | South America | 5 | 1 | 6 | 0.96 | 0.86 |
| *Alternanthera pungens* | Amaranthaceae | Herb | Perennial | Tropical America | 6 | 1 | 7 | 1 | 1 |
| *Ambrosia artemisiifolia* | Asteraceae | Shrub | Annual | North America | 1636 | 545 | 2181 | 0.9 | 0.9 |
| *Anthemis cotula* | Asteraceae | Herb | Annual | Temperate Eurasia | 2761 | 920 | 3681 | 0.86 | 0.86 |
| *Argemone mexicana* | Papaveraceae | Herb | Annual | West Indies | 29 | 9 | 38 | 0.98 | 0.89 |
| *Asclepias curassavica* | Asclepiadaceae | Herb | Perennial | Tropical America | 8 | 2 | 10 | 0.95 | 0.84 |
| *Asphodelus tenuifolius* | Asphodelaceae | Herb | Annual | Tropical Africa & West Asia | 122 | 40 | 162 | 0.99 | 0.99 |
| *Bacopa monnieri* | Scrophulariaceae | Herb | Perennial | Tropical America | 8 | 2 | 10 | 0.99 | 0.96 |
| *Bidens pilosa* | Asteraceae | Herb | Annual | South America | 44 | 14 | 58 | 0.97 | 0.96 |
| *Capsella bursa-pastoris* | Cruciferae | Herb | Biennial | Temperate Europe | 8427 | 2809 | 11236 | 0.75 | 0.75 |
| *Cardamine hirsuta* | Cruciferae | Herb | Annual | Temperate Eurasia | 10158 | 3386 | 13544 | 0.73 | 0.73 |
| *Cardiospermum halicacabum* | Sapindaceae | Climber | Perennial | South America | 8 | 2 | 10 | 0.93 | 0.97 |
| *Celosia argentea* | Amaranthaceae | Herb | Annual | Tropical America | 6 | 1 | 7 | 0.87 | 0.28 |
| *Ceratophyllum demersum* | Ceratophyllaceae | Herb | Perennial | Tropical America | 5540 | 1846 | 7386 | 0.8 | 0.81 |
| *Chenopodium album* | Chenopodiaceae | Herb | Annual | Paleotropical | 8201 | 2733 | 10934 | 0.76 | 0.76 |
| *Chloris barbata* | Poaceae | Grass | Annual | Tropical America & Africa | 3 | 1 | 4 | 0.95 | 1 |
| *Chrozophora plicata* | Euphorbiacaea | Herb | Annual | Tropical Africa | 56 | 18 | 74 | 0.99 | 0.99 |
| *Convolvulus arvensis* | Convolvulaceae | Climber | Perennial | Temperate Eurasia | 8619 | 2873 | 11492 | 0.75 | 0.75 |
| *Conyza bonariensis* | Asteraceae | Herb | Annual | West Asia | 519 | 172 | 691 | 0.96 | 0.95 |
| *Corchorus olitorius* | Tiliaceae | Herb | Annual | Tropical Africa | 3 | 1 | 4 | 0.99 | 0.98 |
| *Coronopus didymus* | Cruciferae | Herb | Biennial | Tropical America | 2796 | 932 | 3728 | 0.88 | 0.87 |
| *Cyclospermum leptophyllum* | Umbelliferae | Herb | Annual | Tropical America | 8 | 2 | 10 | 0.9 | 0.84 |
| *Cyperus difformis* | Cyperaceae | Sedge | Annual | Tropical America | 49 | 16 | 65 | 0.99 | 0.99 |
| *Cyperus rotundus* | Cyperaceae | Sedge | Annual | Paleotropical | 186 | 62 | 248 | 0.98 | 0.97 |
| *Datura inoxia* | Solanaceae | Shrub | Annual | South America | 33 | 10 | 43 | 0.99 | 0.96 |
| *Datura stramonium* | Solanaceae | Shrub | Annual | Tropical America | 3537 | 1179 | 4716 | 0.84 | 0.84 |
| *Daucus carota* | Umbelliferae | Herb | Biennial | Temperate Eurasia | 7345 | 2448 | 9793 | 0.77 | 0.77 |
| *Dysphania ambrosioides* | Caryophyllaceae | Herb | Perennial | Tropical America | 9 | 2 | 11 | 0.87 | 0.8 |
| *Echinochloa colona* | Poaceae | Grass | Annual | Paleotropical | 78 | 25 | 103 | 0.97 | 0.96 |
| *Echinochloa crus-galli* | Poaceae | Grass | Annual | Paleotropical | 7075 | 2358 | 9433 | 0.77 | 0.77 |
| *Euphorbia brasiliensis* | Euphorbiacaea | Herb | Annual | South America | 488 | 162 | 650 | 0.97 | 0.96 |
| *Euphorbia helioscopia* | Euphorbiacaea | Herb | Annual | West Asia | 12066 | 4021 | 16087 | 0.7 | 0.71 |
| *Euphorbia heterophylla* | Euphorbiacaea | Herb | Annual | Mexico | 3 | 1 | 4 | 0.98 | 0.1 |
| *Euphorbia hirta* | Euphorbiacaea | Herb | Annual | Tropical America | 3 | 1 | 4 | 0.93 | 0.99 |
| *Galinsoga parviflora* | Asteraceae | Herb | Annual | Tropical America | 3645 | 1215 | 4860 | 0.85 | 0.84 |
| *Gamochaeta coarctata* | Asteraceae | Herb | Perennial | South America | 8 | 2 | 10 | 0.97 | 0.96 |
| *Gamochaeta pensylvanica* | Asteraceae | Herb | Annual | South America | 6 | 1 | 7 | 0.99 | 0.86 |
| *Gnaphalium purpureum* | Asteraceae | Herb | Annual | Tropical America | 9 | 2 | 11 | 0.92 | 0.85 |
| *Impatiens balsamina* | Balsaminaceae | Herb | Annual | Southeast Asia | 18 | 5 | 23 | 0.96 | 0.94 |
| *Ipomoea indica* | Convolvulaceae | Climber | Perennial | South America | 58 | 19 | 77 | 0.99 | 0.99 |
| *Lantana camara* | Verbenaceae | Shrub | Perennial | Tropical America | 9 | 3 | 12 | 0.97 | 0.87 |
| *Mirabilis jalapa* | Nyctaginaceae | Herb | Perennial | Tropical America | 54 | 18 | 72 | 0.95 | 0.95 |
| *Nicandra physalodes* | Loganiaceae | Herb | Annual | Peru | 855 | 285 | 1140 | 0.93 | 0.93 |
| *Oxalis corymbosa* | Geraniaceae | Herb | Perennial | South America | 27 | 9 | 36 | 0.97 | 0.96 |
| *Oxalis debilis* | Geraniaceae | Herb | Perennial | South America | 145 | 48 | 193 | 0.99 | 0.98 |
| *Oxalis pes-caprae* | Geraniaceae | Herb | Perennial | South Africa | 164 | 54 | 218 | 0.98 | 0.97 |
| *Phragmites australis* | Poaceae | Grass | Perennial | Temperate Eurasia | 6001 | 2000 | 8001 | 0.79 | 0.79 |
| *Pistia stratiotes* | Araceae | Herb | Perennial | North America | 26 | 8 | 34 | 0.97 | 0.96 |
| *Polypogon monspeliensis* | Poaceae | Grass | Annual | Temperate Eurasia | 588 | 196 | 784 | 0.94 | 0.94 |
| *Potamogeton crispus* | Haloragaceae | Herb | Perennial | Temperate Europe | 4881 | 1626 | 6507 | 0.82 | 0.81 |
| *Ricinus communis* | Euphorbiacaea | Tree | Perennial | Tropical Africa | 120 | 40 | 160 | 0.97 | 0.94 |
| *Senna obtusifolia* | Leguminosae | Shrub | Annual | Tropical America | 6 | 2 | 8 | 0.99 | 0.98 |
| *Solanum americanum* | Solanaceae | Herb | Perennial | North & South America | 18 | 6 | 24 | 0.98 | 0.97 |
| *Tribulus terrestris* | Zygophyllaceae | Herb | Perennial | Pantropical | 207 | 68 | 275 | 0.98 | 0.97 |
| *Urochloa panicoides* | Poaceae | Grass | Annual | Tropical Africa | 10 | 3 | 13 | 0.99 | 0.99 |
| *Xanthium strumarium* | Asteraceae | Herb | Annual | South America | 779 | 259 | 1038 | 0.93 | 0.92 |
| North America |  |  |  |  |  |  |  |  |  |
| *Acacia farnesiana* | Leguminosae | Shrub | Perennial | Tropical America | 518 | 221 | 739 | 0.95 | 0.94 |
| *Acanthospermum hispidium* | Asteraceae | Herb | Annual | Tropical America | 47 | 20 | 67 | 0.99 | 0.97 |
| *Achyranthes aspera* | Amaranthaceae | Herb | Perennial | Tropical America | 119 | 51 | 170 | 0.99 | 0.98 |
| *Acmella radicans* | Asteraceae | Herb | Annual | South America | 112 | 48 | 160 | 0.99 | 0.98 |
| *Aeschynomene americana* | Leguminosae | Herb | Perennial | Tropical America | 306 | 131 | 437 | 0.97 | 0.96 |
| *Aeschynomene indica* | Leguminosae | Herb | Annual | North & Central America | 84 | 36 | 120 | 0.99 | 0.99 |
| *Ageratum conyzoides* | Asteraceae | Herb | Annual | South America | 108 | 46 | 154 | 0.99 | 0.97 |
| *Alternanthera paronychioides* | Amaranthaceae | Herb | Perennial | South America & West Indies | 10 | 3 | 13 | 0.99 | 0.85 |
| *Alternanthera philoxeroides* | Amaranthaceae | Herb | Perennial | South America | 609 | 260 | 869 | 0.96 | 0.95 |
| *Alternanthera pungens* | Amaranthaceae | Herb | Perennial | Tropical America | 57 | 24 | 81 | 0.98 | 0.95 |
| *Alternanthera sessilis* | Amaranthaceae | Herb | Perennial | Tropical America | 51 | 21 | 72 | 0.98 | 0.97 |
| *Alternanthera tenella* | Amaranthaceae | Herb | Perennial | South America | 11 | 4 | 15 | 0.96 | 0.95 |
| *Ambrosia artemisiifolia* | Asteraceae | Shrub | Annual | North America | 1653 | 708 | 2361 | 0.84 | 0.83 |
| *Anthemis cotula* | Asteraceae | Herb | Annual | Temperate Eurasia | 1927 | 825 | 2752 | 0.82 | 0.81 |
| *Antigonon leptopus* | Polygonaceae | Climber | Perennial | South America | 198 | 84 | 282 | 0.97 | 0.97 |
| *Argemone mexicana* | Papaveraceae | Herb | Annual | West Indies | 224 | 95 | 319 | 0.96 | 0.93 |
| *Asclepias curassavica* | Asclepiadaceae | Herb | Perennial | Tropical America | 1109 | 474 | 1583 | 0.93 | 0.93 |
| *Bacopa monnieri* | Scrophulariaceae | Herb | Perennial | Tropical America | 315 | 134 | 449 | 0.96 | 0.95 |
| *Bidens pilosa* | Asteraceae | Herb | Annual | South America | 808 | 345 | 1153 | 0.94 | 0.93 |
| *Calotropis gigantea* | Asclepiadaceae | Shrub | Perennial | Tropical Africa | 3 | 1 | 4 | 0.99 | 0.99 |
| *Calotropis procera* | Asclepiadaceae | Shrub | Perennial | Tropical Africa | 68 | 29 | 97 | 0.99 | 0.99 |
| *Capsella bursa-pastoris* | Cruciferae | Herb | Biennial | Temperate Europe | 1762 | 754 | 2516 | 0.83 | 0.8 |
| *Cardamine hirsuta* | Cruciferae | Herb | Annual | Temperate Eurasia | 573 | 245 | 818 | 0.94 | 0.93 |
| *Cardiospermum halicacabum* | Sapindaceae | Climber | Perennial | South America | 361 | 154 | 515 | 0.95 | 0.94 |
| *Celosia argentea* | Amaranthaceae | Herb | Annual | Tropical America | 44 | 18 | 62 | 0.96 | 0.94 |
| *Ceratophyllum demersum* | Ceratophyllaceae | Herb | Perennial | Tropical America | 1128 | 483 | 1611 | 0.85 | 0.82 |
| *Chamaecrista absus* | Leguminosae | Herb | Annual | Pantropical | 44 | 18 | 62 | 0.99 | 0.99 |
| *Chamaecrista rotundifolia* | Leguminosae | Herb | Annual | Tropical America | 56 | 23 | 79 | 0.99 | 0.99 |
| *Chenopodium album* | Chenopodiaceae | Herb | Annual | Paleotropical | 1463 | 627 | 2090 | 0.83 | 0.82 |
| *Chloris barbata* | Poaceae | Grass | Annual | Tropical America & Africa | 85 | 36 | 121 | 0.99 | 0.97 |
| *Chromolaena odorata* | Asteraceae | Shrub | Perennial | Central America | 388 | 165 | 553 | 0.97 | 0.96 |
| *Cissampelos pareira* | Menispermaceae | Climber | Perennial | South America | 404 | 172 | 576 | 0.97 | 0.96 |
| *Cleome gynandra* | Capparaceae | Herb | Annual | Paleotropical | 59 | 25 | 84 | 0.98 | 0.98 |
| *Cleome rutidosperma* | Capparaceae | Herb | Annual | Tropical Africa | 10 | 3 | 13 | 0.99 | 0.98 |
| *Clidemia hirta* | Melastomataceae | Shrub | Perennial | Tropical America | 116 | 49 | 165 | 0.99 | 0.98 |
| *Convolvulus arvensis* | Convolvulaceae | Climber | Perennial | Temperate Eurasia | 1342 | 575 | 1917 | 0.84 | 0.82 |
| *Conyza bonariensis* | Asteraceae | Herb | Annual | West Asia | 206 | 88 | 294 | 0.96 | 0.94 |
| *Corchorus aestuans* | Tiliaceae | Herb | Perennial | Tropical America | 46 | 19 | 65 | 0.99 | 0.97 |
| *Coronopus didymus* | Cruciferae | Herb | Biennial | Tropical America | 266 | 113 | 379 | 0.95 | 0.95 |
| *Crassocephalum crepidioides* | Asteraceae | Herb | Annual | Tropical Africa | 5 | 2 | 7 | 0.94 | 0.94 |
| *Cyclospermum leptophyllum* | Umbelliferae | Herb | Annual | Tropical America | 357 | 153 | 510 | 0.96 | 0.94 |
| *Cyperus difformis* | Cyperaceae | Sedge | Annual | Tropical America | 80 | 34 | 114 | 0.97 | 0.95 |
| *Cyperus iria* | Cyperaceae | Sedge | Annual | Paleotropical | 322 | 138 | 460 | 0.96 | 0.95 |
| *Cyperus rotundus* | Cyperaceae | Sedge | Annual | Paleotropical | 390 | 167 | 557 | 0.94 | 0.9 |
| *Datura inoxia* | Solanaceae | Shrub | Annual | South America | 182 | 78 | 260 | 0.97 | 0.94 |
| *Datura stramonium* | Solanaceae | Shrub | Annual | Tropical America | 1056 | 452 | 1508 | 0.89 | 0.86 |
| *Daucus carota* | Umbelliferae | Herb | Biennial | Temperate Eurasia | 1256 | 537 | 1793 | 0.89 | 0.88 |
| *Desmanthus virgatus* | Leguminosae | Shrub | Perennial | Tropical America | 194 | 83 | 277 | 0.97 | 0.97 |
| *Dysphania ambrosioides* | Caryophyllaceae | Herb | Perennial | Tropical America | 48 | 20 | 68 | 0.98 | 0.95 |
| *Echinochloa colona* | Poaceae | Grass | Annual | Paleotropical | 625 | 267 | 892 | 0.91 | 0.9 |
| *Echinochloa crus-galli* | Poaceae | Grass | Annual | Paleotropical | 1909 | 818 | 2727 | 0.81 | 0.79 |
| *Emilia sonchifolia* | Asteraceae | Herb | Annual | Tropical Africa | 65 | 27 | 92 | 0.98 | 0.97 |
| *Euphorbia brasiliensis* | Euphorbiacaea | Herb | Annual | South America | 437 | 186 | 623 | 0.94 | 0.93 |
| *Euphorbia cyathophora* | Euphorbiacaea | Herb | Annual | North & South America | 469 | 200 | 669 | 0.92 | 0.9 |
| *Euphorbia helioscopia* | Euphorbiacaea | Herb | Annual | West Asia | 94 | 40 | 134 | 0.95 | 0.92 |
| *Euphorbia heterophylla* | Euphorbiacaea | Herb | Annual | Mexico | 535 | 228 | 763 | 0.95 | 0.94 |
| *Euphorbia hirta* | Euphorbiacaea | Herb | Annual | Tropical America | 359 | 153 | 512 | 0.96 | 0.96 |
| *Euphorbia thymifolia* | Euphorbiacaea | Herb | Annual | Tropical America | 78 | 33 | 111 | 0.98 | 0.97 |
| *Evolvulus nummularius* | Convolvulaceae | Herb | Annual | West Indies | 41 | 17 | 58 | 0.98 | 0.97 |
| *Galinsoga parviflora* | Asteraceae | Herb | Annual | Tropical America | 559 | 239 | 798 | 0.94 | 0.92 |
| *Gamochaeta coarctata* | Asteraceae | Herb | Perennial | South America | 18 | 7 | 25 | 0.98 | 0.96 |
| *Gamochaeta pensylvanica* | Asteraceae | Herb | Annual | South America | 156 | 66 | 222 | 0.97 | 0.96 |
| *Gnaphalium polycaulon* | Asteraceae | Herb | Annual | West Indies | 5 | 2 | 7 | 0.99 | 0.93 |
| *Gnaphalium purpureum* | Asteraceae | Herb | Annual | Tropical America | 771 | 330 | 1101 | 0.92 | 0.9 |
| *Gomphrena serrata* | Amaranthaceae | Herb | Annual | Tropical America | 369 | 158 | 527 | 0.97 | 0.96 |
| *Heliotropium indicum* | Boraginaceae | Herb | Annual | South America | 505 | 216 | 721 | 0.94 | 0.93 |
| *Hyptis suaveolens* | Lamiaceae | Herb | Annual | South America | 192 | 81 | 273 | 0.98 | 0.98 |
| *Impatiens balsamina* | Balsaminaceae | Herb | Annual | Southeast Asia | 76 | 32 | 108 | 0.97 | 0.93 |
| *Ipomoea carnea* | Convolvulaceae | Shrub | Perennial | South America | 106 | 45 | 151 | 0.99 | 0.98 |
| *Ipomoea fistulosa* | Convolvulaceae | Shrub | Perennial | South America | 17 | 7 | 24 | 0.99 | 0.98 |
| *Ipomoea hederifolia* | Convolvulaceae | Climber | Annual | Tropical America | 223 | 95 | 318 | 0.97 | 0.95 |
| *Ipomoea indica* | Convolvulaceae | Climber | Perennial | South America | 201 | 86 | 287 | 0.98 | 0.97 |
| *Ipomoea quamoclit* | Convolvulaceae | Climber | Annual | Tropical America | 136 | 57 | 193 | 0.97 | 0.96 |
| *Lantana camara* | Verbenaceae | Shrub | Perennial | Tropical America | 628 | 269 | 897 | 0.95 | 0.93 |
| *Leonotis nepetifolia* | Lamiaceae | Shrub | Annual | Tropical America | 176 | 75 | 251 | 0.97 | 0.95 |
| *Lysiloma latisiliquum* | Fabaceae | Tree | Perennial | Caribbean & Central America | 185 | 79 | 264 | 0.99 | 0.99 |
| *Macroptilium atropurpureum* | Leguminosae | Climber | Perennial | Tropical Central America | 266 | 113 | 379 | 0.97 | 0.97 |
| *Malachra capitata* | Malvaceae | Herb | Annual | Tropical America | 63 | 26 | 89 | 0.98 | 0.97 |
| *Malvastrum coromandelianum* | Malvaceae | Herb | Annual | West Indies | 143 | 61 | 204 | 0.98 | 0.95 |
| *Martynia annua* | Martyniaceae | Herb | Annual | Tropical America | 119 | 50 | 169 | 0.99 | 0.98 |
| *Mecardonia procumbens* | Scrophulariaceae | Herb | Perennial | Tropical America | 274 | 117 | 391 | 0.96 | 0.95 |
| *Merremia aegyptia* | Convolvulaceae | Climber | Perennial | Pantropical | 87 | 37 | 124 | 0.99 | 0.99 |
| *Mikania micrantha* | Asteraceae | Climber | Annual | Tropical America | 156 | 66 | 222 | 0.98 | 0.98 |
| *Mimosa pudica* | Leguminosae | Herb | Perennial | South America | 185 | 78 | 263 | 0.98 | 0.97 |
| *Mirabilis jalapa* | Nyctaginaceae | Herb | Perennial | Tropical America | 326 | 139 | 465 | 0.95 | 0.93 |
| *Monochoria vaginalis* | Pontederiaceae | Herb | Perennial | Southeast Asia | 6 | 2 | 8 | 0.99 | 1 |
| *Nicandra physalodes* | Loganiaceae | Herb | Annual | Peru | 238 | 101 | 339 | 0.95 | 0.92 |
| *Nicotiana plumbaginifolia* | Solanaceae | Herb | Annual | Tropical America | 49 | 20 | 69 | 0.99 | 0.97 |
| *Opuntia dillenii* | Cactaceae | Cactus | Perennial | Mexico | 20 | 8 | 28 | 0.99 | 1 |
| *Opuntia stricta* | Cactaceae | Cactus | Perennial | Mexico | 36 | 15 | 51 | 0.99 | 0.98 |
| *Oxalis debilis* | Geraniaceae | Herb | Perennial | South America | 54 | 23 | 77 | 0.99 | 0.97 |
| *Oxalis pes-caprae* | Geraniaceae | Herb | Perennial | South Africa | 34 | 14 | 48 | 0.99 | 0.97 |
| *Pennisetum purpureum* | Poaceae | Grass | Perennial | Tropical Africa | 162 | 69 | 231 | 0.98 | 0.97 |
| *Phragmites australis* | Poaceae | Grass | Perennial | Temperate Eurasia | 1530 | 655 | 2185 | 0.83 | 0.82 |
| *Physalis minima* | Solanaceae | Herb | Annual | Pantropical | 41 | 17 | 58 | 0.98 | 0.96 |
| *Physalis pruinosa* | Solanaceae | Herb | Annual | Mexico | 24 | 9 | 33 | 0.98 | 0.97 |
| *Pilea microphylla* | Urticaceae | Herb | Perennial | Tropical America | 180 | 76 | 256 | 0.97 | 0.96 |
| *Pistia stratiotes* | Araceae | Herb | Perennial | North America | 434 | 186 | 620 | 0.96 | 0.95 |
| *Polypogon monspeliensis* | Poaceae | Grass | Annual | Temperate Eurasia | 843 | 361 | 1204 | 0.88 | 0.87 |
| *Portulaca quadrifida* | Portulacaceae | Herb | Annual | Pantropical | 4 | 1 | 5 | 1 | 0.99 |
| *Potamogeton crispus* | Haloragaceae | Herb | Perennial | Temperate Europe | 1191 | 510 | 1701 | 0.9 | 0.88 |
| *Prosopis juliflora* | Leguminosae | Tree | Perennial | Mexico & Central America | 103 | 44 | 147 | 0.99 | 0.98 |
| *Ricinus communis* | Euphorbiacaea | Tree | Perennial | Tropical Africa | 236 | 101 | 337 | 0.95 | 0.93 |
| *Ruellia tuberosa* | Acanthaceae | Herb | Perennial | Tropical America | 27 | 11 | 38 | 0.99 | 0.97 |
| *Salvinia molesta* | Salviniaceae | Herb | Perennial | South America | 131 | 56 | 187 | 0.98 | 0.98 |
| *Scoparia dulcis* | Scrophulariaceae | Herb | Annual | South America | 129 | 55 | 184 | 0.98 | 0.96 |
| *Senna alata* | Leguminosae | Shrub | Perennial | South America | 87 | 36 | 123 | 0.97 | 0.96 |
| *Senna hirsuta* | Leguminosae | Herb | Perennial | Tropical America | 229 | 98 | 327 | 0.98 | 0.96 |
| *Senna obtusifolia* | Leguminosae | Shrub | Annual | Tropical America | 409 | 175 | 584 | 0.94 | 0.93 |
| *Senna occidentalis* | Leguminosae | Shrub | Perennial | South America | 360 | 154 | 514 | 0.95 | 0.95 |
| *Senna uniflora* | Leguminosae | Herb | Annual | Tropical America | 204 | 87 | 291 | 0.98 | 0.98 |
| *Sida acuta* | Malvaceae | Shrub | Perennial | Pantropical | 227 | 96 | 323 | 0.98 | 0.96 |
| *Solanum americanum* | Solanaceae | Herb | Perennial | North & South America | 586 | 250 | 836 | 0.92 | 0.9 |
| *Solanum seaforthianum* | Solanaceae | Climber | Perennial | Tropical America | 76 | 32 | 108 | 0.98 | 0.96 |
| *Solanum torvum* | Solanaceae | Shrub | Perennial | Tropical America | 345 | 147 | 492 | 0.97 | 0.96 |
| *Solanum viarum* | Solanaceae | Shrub | Perennial | South America | 22 | 9 | 31 | 0.99 | 0.99 |
| *Spermacoce latifolia* | Rubiaceae | Herb | Perennial | South America | 10 | 3 | 13 | 0.97 | 0.96 |
| *Stachytarpheta jamaicensis* | Verbenaceae | Shrub | Perennial | South America | 98 | 42 | 140 | 0.99 | 0.97 |
| *Stachytarpheta urticifolia* | Verbenaceae | Shrub | Perennial | Tropical America | 78 | 33 | 111 | 0.99 | 0.98 |
| *Synedrella nodiflora* | Asteraceae | Herb | Annual | Tropical America | 100 | 42 | 142 | 0.99 | 0.97 |
| *Tribulus terrestris* | Zygophyllaceae | Herb | Perennial | Pantropical | 728 | 312 | 1040 | 0.9 | 0.86 |
| *Tridax procumbens* | Asteraceae | Herb | Perennial | Mexico | 227 | 96 | 323 | 0.97 | 0.96 |
| *Triumfetta rhomboidea* | Tiliaceae | Shrub | Perennial | Tropical America | 4 | 1 | 5 | 0.99 | 1 |
| *Turnera ulmifolia* | Turneraceae | Shrub | Perennial | Tropical America | 65 | 27 | 92 | 0.98 | 0.98 |
| *Urena lobata* | Malvaceae | Herb | Perennial | Tropical Africa | 64 | 27 | 91 | 0.99 | 0.99 |
| *Urochloa panicoides* | Poaceae | Grass | Annual | Tropical Africa | 19 | 7 | 26 | 0.99 | 0.98 |
| *Waltheria indica* | Sterculiaceae | Shrub | Perennial | Tropical America | 213 | 90 | 303 | 0.97 | 0.96 |
| *Xanthium strumarium* | Asteraceae | Herb | Annual | South America | 1137 | 487 | 1624 | 0.84 | 0.82 |
| *Youngia japonica* | Asteraceae | Herb | Biennial | South America & Australia | 119 | 50 | 169 | 0.98 | 0.97 |
| South America |  |  |  |  |  |  |  |  |  |
| *Acacia farnesiana* | Leguminosae | Shrub | Perennial | Tropical America | 105 | 45 | 150 | 0.97 | 0.89 |
| *Acanthospermum hispidium* | Asteraceae | Herb | Annual | Tropical America | 85 | 36 | 121 | 0.95 | 0.83 |
| *Achyranthes aspera* | Amaranthaceae | Herb | Perennial | Tropical America | 72 | 30 | 102 | 0.95 | 0.94 |
| *Acmella radicans* | Asteraceae | Herb | Annual | South America | 65 | 27 | 92 | 0.99 | 0.98 |
| *Adenostemma lavenia* | Caryophyllaceae | Herb | Perennial | South America | 7 | 2 | 9 | 0.94 | 0.59 |
| *Aeschynomene americana* | Leguminosae | Herb | Perennial | Tropical America | 276 | 117 | 393 | 0.94 | 0.9 |
| *Ageratum conyzoides* | Asteraceae | Herb | Annual | South America | 287 | 123 | 410 | 0.93 | 0.9 |
| *Alternanthera paronychioides* | Amaranthaceae | Herb | Perennial | South America & West Indies | 33 | 13 | 46 | 0.93 | 0.73 |
| *Alternanthera philoxeroides* | Amaranthaceae | Herb | Perennial | South America | 30 | 12 | 42 | 0.95 | 0.89 |
| *Alternanthera pungens* | Amaranthaceae | Herb | Perennial | Tropical America | 43 | 18 | 61 | 0.95 | 0.87 |
| *Alternanthera sessilis* | Amaranthaceae | Herb | Perennial | Tropical America | 77 | 33 | 110 | 0.95 | 0.91 |
| *Alternanthera tenella* | Amaranthaceae | Herb | Perennial | South America | 63 | 27 | 90 | 0.91 | 0.78 |
| *Ambrosia artemisiifolia* | Asteraceae | Shrub | Annual | North America | 63 | 26 | 89 | 0.92 | 0.96 |
| *Anthemis cotula* | Asteraceae | Herb | Annual | Temperate Eurasia | 406 | 174 | 580 | 0.87 | 0.81 |
| *Antigonon leptopus* | Polygonaceae | Climber | Perennial | South America | 58 | 24 | 82 | 0.97 | 0.85 |
| *Argemone mexicana* | Papaveraceae | Herb | Annual | West Indies | 47 | 19 | 66 | 0.94 | 0.9 |
| *Asclepias curassavica* | Asclepiadaceae | Herb | Perennial | Tropical America | 417 | 178 | 595 | 0.92 | 0.88 |
| *Bacopa monnieri* | Scrophulariaceae | Herb | Perennial | Tropical America | 59 | 24 | 83 | 0.93 | 0.85 |
| *Bidens pilosa* | Asteraceae | Herb | Annual | South America | 292 | 124 | 416 | 0.92 | 0.86 |
| *Calotropis gigantea* | Asclepiadaceae | Shrub | Perennial | Tropical Africa | 6 | 2 | 8 | 0.9 | 0.98 |
| *Calotropis procera* | Asclepiadaceae | Shrub | Perennial | Tropical Africa | 49 | 20 | 69 | 0.97 | 0.87 |
| *Capsella bursa-pastoris* | Cruciferae | Herb | Biennial | Temperate Europe | 64 | 27 | 91 | 0.94 | 0.88 |
| *Cardamine hirsuta* | Cruciferae | Herb | Annual | Temperate Eurasia | 34 | 14 | 48 | 0.98 | 0.96 |
| *Cardiospermum halicacabum* | Sapindaceae | Climber | Perennial | South America | 82 | 35 | 117 | 0.9 | 0.79 |
| *Celosia argentea* | Amaranthaceae | Herb | Annual | Tropical America | 24 | 9 | 33 | 0.91 | 0.79 |
| *Ceratophyllum demersum* | Ceratophyllaceae | Herb | Perennial | Tropical America | 18 | 7 | 25 | 0.94 | 0.8 |
| *Chamaecrista absus* | Leguminosae | Herb | Annual | Pantropical | 7 | 2 | 9 | 0.76 | 0.71 |
| *Chamaecrista rotundifolia* | Leguminosae | Herb | Annual | Tropical America | 179 | 76 | 255 | 0.91 | 0.85 |
| *Chenopodium album* | Chenopodiaceae | Herb | Annual | Paleotropical | 24 | 9 | 33 | 0.98 | 0.91 |
| *Chloris barbata* | Poaceae | Grass | Annual | Tropical America & Africa | 96 | 40 | 136 | 0.97 | 0.9 |
| *Chromolaena odorata* | Asteraceae | Shrub | Perennial | Central America | 298 | 127 | 425 | 0.94 | 0.87 |
| *Cissampelos pareira* | Menispermaceae | Climber | Perennial | South America | 359 | 153 | 512 | 0.92 | 0.9 |
| *Cleome rutidosperma* | Capparaceae | Herb | Annual | Tropical Africa | 9 | 3 | 12 | 0.98 | 0.99 |
| *Clidemia hirta* | Melastomataceae | Shrub | Perennial | Tropical America | 270 | 115 | 385 | 0.92 | 0.86 |
| *Convolvulus arvensis* | Convolvulaceae | Climber | Perennial | Temperate Eurasia | 28 | 11 | 39 | 0.97 | 0.94 |
| *Conyza bonariensis* | Asteraceae | Herb | Annual | West Asia | 231 | 98 | 329 | 0.91 | 0.85 |
| *Corchorus aestuans* | Tiliaceae | Herb | Perennial | Tropical America | 31 | 12 | 43 | 0.95 | 0.98 |
| *Coronopus didymus* | Cruciferae | Herb | Biennial | Tropical America | 51 | 21 | 72 | 0.94 | 0.89 |
| *Crassocephalum crepidioides* | Asteraceae | Herb | Annual | Tropical Africa | 12 | 5 | 17 | 0.98 | 0.97 |
| *Croton bonplandianus* | Euphorbiacaea | Herb | Perennial | South America | 66 | 27 | 93 | 0.98 | 0.98 |
| *Cyclospermum leptophyllum* | Umbelliferae | Herb | Annual | Tropical America | 118 | 50 | 168 | 0.96 | 0.9 |
| *Cyperus difformis* | Cyperaceae | Sedge | Annual | Tropical America | 12 | 5 | 17 | 0.87 | 0.71 |
| *Cyperus iria* | Cyperaceae | Sedge | Annual | Paleotropical | 52 | 22 | 74 | 0.9 | 0.84 |
| *Cyperus rotundus* | Cyperaceae | Sedge | Annual | Paleotropical | 98 | 42 | 140 | 0.93 | 0.9 |
| *Datura inoxia* | Solanaceae | Shrub | Annual | South America | 35 | 15 | 50 | 0.93 | 0.93 |
| *Datura stramonium* | Solanaceae | Shrub | Annual | Tropical America | 49 | 20 | 69 | 0.97 | 0.93 |
| *Daucus carota* | Umbelliferae | Herb | Biennial | Temperate Eurasia | 12 | 5 | 17 | 0.82 | 0.89 |
| *Desmanthus virgatus* | Leguminosae | Shrub | Perennial | Tropical America | 138 | 59 | 197 | 0.95 | 0.87 |
| *Dysphania ambrosioides* | Caryophyllaceae | Herb | Perennial | Tropical America | 45 | 18 | 63 | 0.98 | 0.88 |
| *Echinochloa colona* | Poaceae | Grass | Annual | Paleotropical | 205 | 87 | 292 | 0.93 | 0.88 |
| *Echinochloa crus-galli* | Poaceae | Grass | Annual | Paleotropical | 71 | 30 | 101 | 0.9 | 0.85 |
| *Emilia sonchifolia* | Asteraceae | Herb | Annual | Tropical Africa | 79 | 33 | 112 | 0.95 | 0.92 |
| *Euphorbia brasiliensis* | Euphorbiacaea | Herb | Annual | South America | 283 | 120 | 403 | 0.92 | 0.89 |
| *Euphorbia cyathophora* | Euphorbiacaea | Herb | Annual | North & South America | 24 | 10 | 34 | 0.96 | 0.69 |
| *Euphorbia helioscopia* | Euphorbiacaea | Herb | Annual | West Asia | 3 | 1 | 4 | 0.89 | 0.62 |
| *Euphorbia heterophylla* | Euphorbiacaea | Herb | Annual | Mexico | 234 | 100 | 334 | 0.93 | 0.88 |
| *Euphorbia hirta* | Euphorbiacaea | Herb | Annual | Tropical America | 274 | 117 | 391 | 0.92 | 0.89 |
| *Euphorbia ovalifolia* | Euphorbiacaea | Herb | Annual | South America | 6 | 2 | 8 | 0.89 | 0.42 |
| *Euphorbia thymifolia* | Euphorbiacaea | Herb | Annual | Tropical America | 129 | 55 | 184 | 0.95 | 0.89 |
| *Evolvulus nummularius* | Convolvulaceae | Herb | Annual | West Indies | 55 | 23 | 78 | 0.93 | 0.87 |
| *Galinsoga parviflora* | Asteraceae | Herb | Annual | Tropical America | 75 | 32 | 107 | 0.93 | 0.9 |
| *Gamochaeta coarctata* | Asteraceae | Herb | Perennial | South America | 68 | 29 | 97 | 0.95 | 0.92 |
| *Gamochaeta pensylvanica* | Asteraceae | Herb | Annual | South America | 59 | 25 | 84 | 0.93 | 0.86 |
| *Gnaphalium polycaulon* | Asteraceae | Herb | Annual | West Indies | 5 | 1 | 6 | 0.94 | 0.21 |
| *Gnaphalium purpureum* | Asteraceae | Herb | Annual | Tropical America | 31 | 13 | 44 | 0.96 | 0.84 |
| *Gomphrena serrata* | Amaranthaceae | Herb | Annual | Tropical America | 130 | 55 | 185 | 0.96 | 0.89 |
| *Heliotropium indicum* | Boraginaceae | Herb | Annual | South America | 269 | 114 | 383 | 0.92 | 0.85 |
| *Hyptis suaveolens* | Lamiaceae | Herb | Annual | South America | 196 | 83 | 279 | 0.95 | 0.91 |
| *Impatiens balsamina* | Balsaminaceae | Herb | Annual | Southeast Asia | 30 | 12 | 42 | 0.96 | 0.93 |
| *Ipomoea carnea* | Convolvulaceae | Shrub | Perennial | South America | 152 | 64 | 216 | 0.95 | 0.87 |
| *Ipomoea fistulosa* | Convolvulaceae | Shrub | Perennial | South America | 4 | 1 | 5 | 0.83 | 0.85 |
| *Ipomoea hederifolia* | Convolvulaceae | Climber | Annual | Tropical America | 131 | 56 | 187 | 0.96 | 0.89 |
| *Ipomoea indica* | Convolvulaceae | Climber | Perennial | South America | 122 | 52 | 174 | 0.98 | 0.94 |
| *Ipomoea quamoclit* | Convolvulaceae | Climber | Annual | Tropical America | 101 | 43 | 144 | 0.94 | 0.88 |
| *Lantana camara* | Verbenaceae | Shrub | Perennial | Tropical America | 301 | 128 | 429 | 0.91 | 0.83 |
| *Leonotis nepetifolia* | Lamiaceae | Shrub | Annual | Tropical America | 61 | 25 | 86 | 0.93 | 0.84 |
| *Macroptilium atropurpureum* | Leguminosae | Climber | Perennial | Tropical Central America | 68 | 28 | 96 | 0.92 | 0.82 |
| *Malachra capitata* | Malvaceae | Herb | Annual | Tropical America | 21 | 9 | 30 | 0.97 | 0.97 |
| *Malvastrum coromandelianum* | Malvaceae | Herb | Annual | West Indies | 82 | 34 | 116 | 0.95 | 0.91 |
| *Martynia annua* | Martyniaceae | Herb | Annual | Tropical America | 54 | 23 | 77 | 0.99 | 0.96 |
| *Mecardonia procumbens* | Scrophulariaceae | Herb | Perennial | Tropical America | 122 | 51 | 173 | 0.95 | 0.93 |
| *Merremia aegyptia* | Convolvulaceae | Climber | Perennial | Pantropical | 77 | 33 | 110 | 0.93 | 0.87 |
| *Mikania micrantha* | Asteraceae | Climber | Annual | Tropical America | 406 | 173 | 579 | 0.91 | 0.84 |
| *Mimosa pudica* | Leguminosae | Herb | Perennial | South America | 203 | 86 | 289 | 0.95 | 0.91 |
| *Mirabilis jalapa* | Nyctaginaceae | Herb | Perennial | Tropical America | 84 | 36 | 120 | 0.9 | 0.88 |
| *Nicandra physalodes* | Loganiaceae | Herb | Annual | Peru | 79 | 33 | 112 | 0.95 | 0.94 |
| *Nicotiana plumbaginifolia* | Solanaceae | Herb | Annual | Tropical America | 17 | 7 | 24 | 0.91 | 0.77 |
| *Opuntia dillenii* | Cactaceae | Cactus | Perennial | Mexico | 4 | 1 | 5 | 1 | 0.99 |
| *Oxalis debilis* | Geraniaceae | Herb | Perennial | South America | 31 | 12 | 43 | 0.95 | 0.96 |
| *Pennisetum purpureum* | Poaceae | Grass | Perennial | Tropical Africa | 145 | 61 | 206 | 0.96 | 0.87 |
| *Phragmites australis* | Poaceae | Grass | Perennial | Temperate Eurasia | 49 | 21 | 70 | 0.94 | 0.76 |
| *Physalis minima* | Solanaceae | Herb | Annual | Pantropical | 35 | 15 | 50 | 0.95 | 0.79 |
| *Physalis pruinosa* | Solanaceae | Herb | Annual | Mexico | 36 | 15 | 51 | 0.98 | 0.95 |
| *Pilea microphylla* | Urticaceae | Herb | Perennial | Tropical America | 171 | 73 | 244 | 0.95 | 0.92 |
| *Pistia stratiotes* | Araceae | Herb | Perennial | North America | 102 | 43 | 145 | 0.93 | 0.89 |
| *Polypogon monspeliensis* | Poaceae | Grass | Annual | Temperate Eurasia | 54 | 23 | 77 | 0.95 | 0.93 |
| *Potamogeton crispus* | Haloragaceae | Herb | Perennial | Temperate Europe | 3 | 1 | 4 | 0.97 | 0.99 |
| *Prosopis juliflora* | Leguminosae | Tree | Perennial | Mexico & Central America | 86 | 36 | 122 | 0.97 | 0.94 |
| *Ricinus communis* | Euphorbiacaea | Tree | Perennial | Tropical Africa | 145 | 61 | 206 | 0.92 | 0.88 |
| *Rorippa dubia* | Cruciferae | Herb | Annual | Tropical America | 10 | 2 | 12 | 0.87 | 0.07 |
| *Ruellia tuberosa* | Acanthaceae | Herb | Perennial | Tropical America | 15 | 6 | 21 | 0.99 | 0.98 |
| *Scoparia dulcis* | Scrophulariaceae | Herb | Annual | South America | 155 | 66 | 221 | 0.91 | 0.85 |
| *Senna alata* | Leguminosae | Shrub | Perennial | South America | 124 | 52 | 176 | 0.94 | 0.84 |
| *Senna hirsuta* | Leguminosae | Herb | Perennial | Tropical America | 114 | 48 | 162 | 0.95 | 0.85 |
| *Senna obtusifolia* | Leguminosae | Shrub | Annual | Tropical America | 194 | 82 | 276 | 0.91 | 0.82 |
| *Senna occidentalis* | Leguminosae | Shrub | Perennial | South America | 217 | 93 | 310 | 0.9 | 0.84 |
| *Senna uniflora* | Leguminosae | Herb | Annual | Tropical America | 51 | 21 | 72 | 0.99 | 0.97 |
| *Sida acuta* | Malvaceae | Shrub | Perennial | Pantropical | 96 | 40 | 136 | 0.93 | 0.83 |
| *Solanum americanum* | Solanaceae | Herb | Perennial | North & South America | 574 | 246 | 820 | 0.88 | 0.87 |
| *Solanum seaforthianum* | Solanaceae | Climber | Perennial | Tropical America | 50 | 21 | 71 | 0.97 | 0.97 |
| *Solanum torvum* | Solanaceae | Shrub | Perennial | Tropical America | 207 | 88 | 295 | 0.98 | 0.96 |
| *Solanum viarum* | Solanaceae | Shrub | Perennial | South America | 44 | 18 | 62 | 0.98 | 0.93 |
| *Spermacoce latifolia* | Rubiaceae | Herb | Perennial | South America | 128 | 54 | 182 | 0.92 | 0.88 |
| *Stachytarpheta jamaicensis* | Verbenaceae | Shrub | Perennial | South America | 77 | 32 | 109 | 0.99 | 0.98 |
| *Stachytarpheta urticifolia* | Verbenaceae | Shrub | Perennial | Tropical America | 270 | 115 | 385 | 0.9 | 0.83 |
| *Synedrella nodiflora* | Asteraceae | Herb | Annual | Tropical America | 162 | 69 | 231 | 0.96 | 0.93 |
| *Tribulus terrestris* | Zygophyllaceae | Herb | Perennial | Pantropical | 28 | 11 | 39 | 0.97 | 0.93 |
| *Tridax procumbens* | Asteraceae | Herb | Perennial | Mexico | 129 | 54 | 183 | 0.97 | 0.95 |
| *Triumfetta rhomboidea* | Tiliaceae | Shrub | Perennial | Tropical America | 16 | 6 | 22 | 0.91 | 0.94 |
| *Turnera subulata* | Turneraceae | Herb | Perennial | Tropical America | 28 | 12 | 40 | 0.91 | 0.85 |
| *Turnera ulmifolia* | Turneraceae | Shrub | Perennial | Tropical America | 49 | 21 | 70 | 0.94 | 0.86 |
| *Urena lobata* | Malvaceae | Herb | Perennial | Tropical Africa | 133 | 57 | 190 | 0.95 | 0.91 |
| *Urochloa panicoides* | Poaceae | Grass | Annual | Tropical Africa | 17 | 6 | 23 | 0.99 | 0.99 |
| *Waltheria indica* | Sterculiaceae | Shrub | Perennial | Tropical America | 115 | 49 | 164 | 0.95 | 0.89 |
| *Xanthium strumarium* | Asteraceae | Herb | Annual | South America | 5 | 2 | 7 | 0.9 | 0.66 |
| *Youngia japonica* | Asteraceae | Herb | Biennial | South America & Australia | 23 | 9 | 32 | 1 | 0.96 |
