# Supplementary material for: Modelling Hotspots for Invasive Alien Plants in India
Source: PLoS One. 2015 Jul 31;10(7):e0134665. doi: 10.1371/journal.pone.0134665 (PMC4521859; doi:10.1371/journal.pone.0134665)
Supplement: S2 Table — The values in the table represent the number of pixels (size: ≈ 4 km) under each class. (DOCX) [file pone.0134665.s006.docx]

**S2 Table**. **Distribution of model consensus classes in the ecoregions of India.** The values in the table represent the number of pixels (size: ≈ 4 km) under each class.

| **Biome type** | **Ecoregions** | **Model consensus classes** | | | |
| --- | --- | --- | --- | --- | --- |
|  |  | **High** | **Medium** | **Low** | **Very low** |
| Deserts and Xeric Shrublands | Deccan Thorn Scrub Forests | 3708 | 3180 | 8717 | 2146 |
|  | Northwestern Thorn Scrub Forests | 843 | 1151 | 4735 | 7003 |
|  | Thar Desert |  |  | 98 | 8920 |
| Flooded Grasslands and Savannas | Rann of Kutch Seasonal Salt Marsh |  |  | 59 | 1133 |
| Mangroves | Godavari-Krishna Mangroves | 326 |  |  |  |
|  | Indus River Delta-Arabian Sea Mangroves | 13 | 11 | 15 | 1 |
|  | Sundarbans Mangroves | 189 |  |  |  |
| Montane Grasslands and Shrublands | Central Tibetan Plateau Alpine Steppe |  |  |  | 837 |
|  | Eastern Himalayan Alpine Shrub and Meadows | 1 | 13 | 144 | 504 |
|  | Karakoram-West Tibetan Plateau Alpine Steppe |  |  |  | 6406 |
|  | North Tibetan Plateau-Kunlun Mountains Alpine Desert |  |  |  | 658 |
|  | Northwestern Himalayan Alpine Shrub and Meadows |  | 3 | 28 | 1862 |
|  | Pamir Alpine Desert and Tundra |  |  |  | 11 |
|  | Western Himalayan Alpine Shrub and Meadows | 1 | 7 | 46 | 718 |
| Rock and Ice | Rock and Ice: Palearctic |  |  |  | 2489 |
| Temperate Broadleaf and Mixed Forests | Eastern Himalayan Broadleaved Forests | 1477 | 610 | 725 | 94 |
|  | Western Himalayan Broadleaved Forests | 293 | 645 | 465 | 1496 |
| Temperate Conifer Forests | Eastern Himalayan Subalpine Conifer Forests | 3 | 51 | 278 | 385 |
|  | Northeastern Himalayan Subalpine Conifer Forests | 5 | 10 | 62 | 202 |
|  | Western Himalayan Subalpine Conifer Forests | 4 | 69 | 132 | 439 |
| Tropical and Subtropical Coniferous Forests | Himalayan Subtropical Pine Forests | 1026 | 966 | 391 | 211 |
|  | Northeast India-Myanmar Pine Forests | 400 | 98 | 11 |  |
| Tropical and Subtropical Dry Broadleaf Forests | Central Deccan Plateau Dry Deciduous Forests | 1611 | 10046 | 1159 | 27 |
|  | Chota-Nagpur Dry Deciduous Forests | 1043 | 4489 | 1226 |  |
|  | East Deccan Dry-Evergreen Forests | 1286 |  |  |  |
|  | Khathiar-Gir Dry Deciduous Forests | 286 | 309 | 7995 | 6161 |
|  | Narmada Valley Dry Deciduous Forests | 149 | 520 | 7466 | 1174 |
|  | Northern Dry Deciduous Forests | 1713 | 1433 |  |  |
|  | South Deccan Plateau Dry Deciduous Forests | 2454 | 1360 | 412 | 12 |
| Tropical and Subtropical Grasslands, Savannas and Shrublands | Terai-Duar Savanna and Grasslands | 235 | 323 | 15 |  |
| Tropical and Subtropical Moist Broadleaf Forests | Andaman Islands Rainforest | 198 | 17 |  |  |
|  | Brahmaputra Valley Semievergreen Forests | 3187 | 1 | 3 |  |
|  | Chin-Hills-Arakan Yoma Montane Forests | 100 |  |  |  |
|  | Eastern Highlands Moist Deciduous Forests | 7445 | 9297 | 1675 | 2 |
|  | Himalayan Subtropical Broad Leaved Forests | 33 | 244 | 6 |  |
|  | Lower Gangetic Plains Moist Deciduous Forests | 2800 | 4316 | 917 |  |
|  | Malabar Coasts Moist Forests | 805 | 678 |  |  |
|  | Meghalaya Subtropical Forests | 2324 | 3 |  |  |
|  | Mizoram-Manipur-Kachin Rainforests | 3083 | 48 | 15 |  |
|  | Nicobar Islands Rainforests |  | 50 | 4 |  |
|  | North Western Ghats Moist Deciduous Forests | 1625 | 731 | 191 |  |
|  | North Western Ghats Montane Rain Forests | 1216 | 410 | 2 |  |
|  | Orissa Semi-Evergreen Forests | 1150 |  |  |  |
|  | South Western Ghats Moist Deciduous Forests | 998 | 232 |  |  |
|  | South Western Ghats Montane Rain Forests | 830 | 334 | 1 |  |
|  | Sunderbans Freshwater Swamp Forests | 370 | 2 |  |  |
|  | Upper Gangetic Forests Moist Deciduous Forests | 40 | 3361 | 8040 | 3491 |
